# Climate‐induced range shifts shaped the present and threaten the future genetic variability of a marine brown alga in the Northwest Pacific

**DOI:** 10.1111/eva.13247

**Published:** 2021-05-18

**Authors:** Xiao‐Han Song, Jorge Assis, Jie Zhang, Xu Gao, Han‐Gil Gao, De‐Lin Duan, Ester A. Serrão, Zi‐Min Hu

**Affiliations:** ^1^ Key Laboratory of Experimental Marine Biology Center for Ocean Mega‐Science Institute of Oceanology Chinese Academy of Sciences Qingdao China; ^2^ Laboratory for Marine Biology and Biotechnology Qingdao National Laboratory for Marine Science and Technology Qingdao China; ^3^ University of Chinese Academy of Sciences Beijing China; ^4^ CCMAR University of Algarve, Campus de Gambelas Faro Portugal; ^5^ Faculty of Biological Science and Research Institute for Basic Science Wonkwang University Iksan Korea; ^6^ Ocean School Yantai University Yantai China

**Keywords:** climate change, genetic diversity, glacial persistence, niche modelling, range shifts, *Sargassum thunbergii*

## Abstract

Glaciation‐induced environmental changes during the last glacial maximum (LGM) have strongly influenced species' distributions and genetic diversity patterns in the northern high latitudes. However, these effects have seldom been assessed on sessile species in the Northwest Pacific. Herein, we chose the brown alga *Sargassum thunbergii* to test this hypothesis, by comparing present population genetic variability with inferred geographical range shifts from the LGM to the present, estimated with species distribution modelling (SDM). Projections for contrasting scenarios of future climate change were also developed to anticipate genetic diversity losses at regional scales. Results showed that *S*. *thunbergii* harbours strikingly rich genetic diversity and multiple divergent lineages in the centre‐northern range of its distribution, in contrast with a poorer genetically distinct lineage in the southern range. SDM hindcasted refugial persistence in the southern range during the LGM as well as post‐LGM expansion of 18 degrees of latitude northward. Approximate Bayesian computation (ABC) analysis further suggested that the multiple divergent lineages in the centre‐northern range limit stem from post‐LGM colonization from the southern survived lineage. This suggests divergence due to demographic bottlenecks during range expansion and massive genetic diversity loss during post‐LGM contraction in the south. The projected future range of *S*. *thunbergii* highlights the threat to unique gene pools that might be lost under global changes.

## 1 INTRODUCTION

In coastal marine environments, climate oscillations during the last glacial maximum (LGM, *c*. 21 thousand years ago [ka]) drove coastline configurations and hence the disjunction of marine glacial refugia (Assis et al., 2017; Hu et al., 2011; Maggs et al., 2008). These refugia are isolated areas uniquely buffered from the drastic fluctuations of glacial climates and expanded to encompass not only past climate shifts, but also contemporary global warming and fluctuating sea levels (Stewart et al., 2010). The unique physical characteristics (e.g. climate dynamics and topography) surrounding climatic refugia enabled ‘relict’ populations to persist and retain important eco‐physiological, phenological and adaptive variability (Morelli et al., 2016). However, the locations of such refugia are insufficiently understood for the northwestern Pacific coasts, where potential ancient populations persisting throughout cold and warm extreme environments may retain higher and unique genetic variability (Assis et al., 2016; Assis, Serrão, et al., 2018). Importantly, populations along species' range edges can have unequal adaptive capabilities to environmental shifts (King et al., 2019). Thus, quantifying evolutionary footprints of past climate‐driven range shifts is key to predict the consequences of future climate scenarios, as threats to rich or endemic gene pools (Assis et al., 2017; Assis, Serrão, et al., 2018).

Coastal brown algae are ideal models to test the causal relationships between genetic diversity patterns and climate‐driven range shifts (Assis, Serrão, et al., 2018). Numerous studies revealed variable roles of climate‐driven range shifts, geographical isolation and genetic bottlenecks in driving phylogeographical structure and lineage differentiation in the Northeast Atlantic (Assis, Serrão, et al., 2018; Neiva et al., 2015; Provan & Maggs, 2012) and Southern Hemisphere (Fraser et al., 2009). The Northwest Pacific, despite being a marine biodiversity hotspot (Briggs & Bowen, 2012) with rich seaweed diversity and endemism (Kerswell, 2006), has not yet been sufficiently studied to answer whether and how species shifted geographical ranges in response to past climate changes. Also, little information resides on the forecasted demographical consequences under future warming scenarios. It is noteworthy that the Northwest Pacific had been heavily influenced by the LGM, profoundly changing sea‐level patterns and the configuration of continental shelves and margins (Wang, 1999), as well as reshaping historical demography and population connectivity (Hu et al., 2011, 2015, 2017). However, no range shift estimates were yet produced, linking present‐day species' genetic variation with spatial distributions across times.

*Sargassum thunbergii* (Mertens ex Roth) Kuntze is a cold‐temperate brown alga endemic to the Northwest Pacific. Historically, this species was common along the entire Pacific coast of China (Tseng, 1983), but southern populations (e.g. latitudes <21°N) have diminished over recent decades (Liu, 2017). The species can drift on the continental shelf west of the Kuroshio Current upon thallus detachment from holdfasts and potentially perform long‐distance dispersal (Qi et al., 2017). Recent phylogeographical studies revealed the role of oceanic currents in driving population genetic connectivity of *S*. *thunbergii* (Li, Hu, Gao, et al., 2017) and identified a north to south genetic break along the coast of China, hypothetically shaped by sea‐level changes during the late Quaternary (Li, Hu, Sun, et al., 2017). To date, no study has addressed the influence of past climate changes on the distribution of genetic pools of *S*. *thunbergii* nor estimated potential genetic losses under contrasting scenarios of future climate change.

The present study addresses two alternative process‐based evolutionary hypotheses for *S*. *thunbergii*, based on both the previously reported patterns of genetic structure, and also on the sharp geographical isolation of the Northwest Pacific marginal seas during the LGM (Wang, 1999): (i) *S*. *thunbergii* persisted in demographically isolated northern and southern refugia in the Northwest Pacific during Quaternary ice ages; (ii) *S*. *thunbergii* survived in southern glacial refugia and expanded northwards following the glaciers retractions, causing homogeneous low diversity regions across the wide northern range and leaving behind most endemic diversity at rear edges. To address these hypotheses, microsatellite markers were used to detect fine‐scale phylogeographical structure and latitudinal diversity patterns along the Northwest Pacific. Additionally, independent species distribution modelling (SDM) (Elith & Leathwick, 2009) and approximate Bayesian computation (ABC) were used to infer potential high‐latitude range expansions and low latitude contractions, from the LGM to the present, including the warmer mid‐Holocene (MH, *c*. 6 ka) period (Mairesse et al., 2013). Together, this information allowed testing whether past climate‐driven edge‐of‐range shifts have influenced present genetic diversity hotspots and if these key areas are at risk of disappearing under projected global warming (Morelli et al., 2016).

## 2 MATERIALS AND METHODS

### 2.1 Study system

**FIGURE 1:**
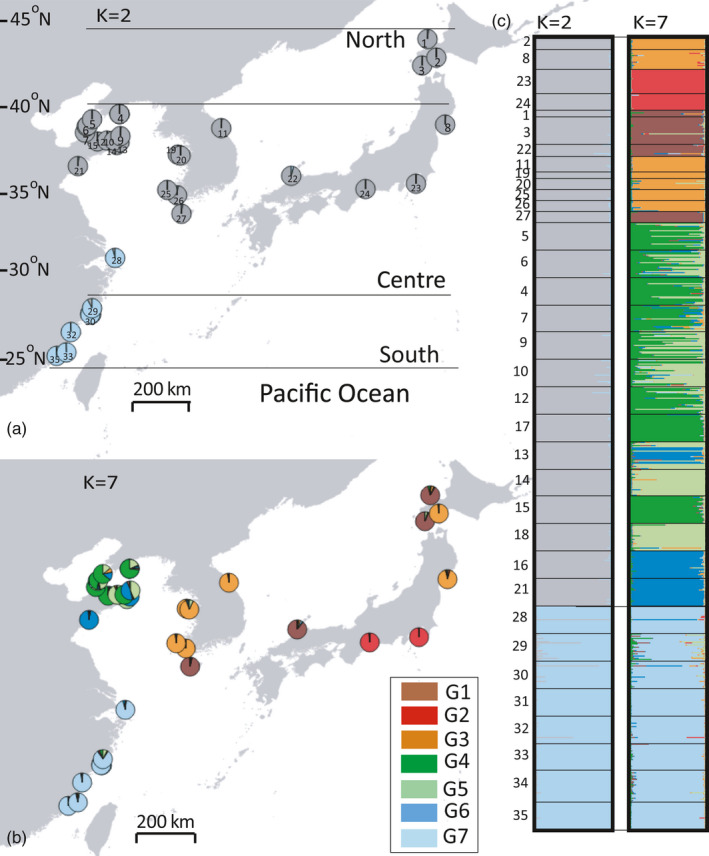
(a) Map of the study area including the latitudinal range limits of *Sargassum thunbergii*. (a–c) Colours depict the genetic subdivision based on structure for the two‐uppermost levels of subdivisions over space (*k* = 2 and *k* = 7). Population numbers (1–35) on the map and plot structure are the same as in Table 1

*Sargassum thunbergii* is found in the Northwest Pacific, as far north as Hokkaido, Japan and south to Hong Kong and Guangdong, China (Tseng, 1983), geographically spanning approximately 22° in latitude (44°–23°N; Figure 1). This species displays marked shore zonation at the intertidal to low supratidal level. It has a perennial holdfast and seasonal variations in growth of stipe, fronds, branches and vesicles, related to temperature shifts in the Northwest Pacific (Koh et al., 1993). Some Sargassaceae in China mature at 19–21°C, reaching a peak in abundance in mid‐May (23.5–25°C), and perishing in late June (27.5–30°C) (Zou et al., 2006). Sexual reproduction is predominant in the population dynamics of *S*. *thunbergii* and usually needs increased resource allocation, which conversely leads to decreased allocation to vegetative regeneration from holdfasts (Chu et al., 2011).

### 2.2 Sampling, genetic diversity and phylogeographical clustering

To characterize current phylogeographical diversity and genetic structuring of *S*. *thunbergii*, populations were sampled throughout its entire distribution in 35 locations between 2013 and 2016 (Figure 1). These locations can be geographically divided into Japan‐Pacific coast, the Sea of Japan, the Yellow‐Bohai Sea and East China Sea, according to different water masses (Table 1). Coastal distances between sampling localities ranged from *c*. 2 to 2779 km. Sampling, specimen preservation and genomic DNA isolation followed Li, Hu, Gao, et al. (2017).

**TABLE 1:** Sites, coordinates and population sizes of *Sthunbergii thunbergii* analysed in this study, and standardized allelic richness ($\hat{\alpha }$), standardized private diversity ($\hat{\pi }$) and gene diversity (*H*_e_) inferred from microsatellites

Sampling site	Water body	Coordinates	*n*	$\hat{\alpha }$	$\hat{\pi }$	*H*_e_
1. Shakotan, Hokkaido, Japan	Sea of Japan	43.36°N, 140.46°E	6	2.60 ± 0.07	0.00 ± 0.00	0.39
2. Muroran, Hokkaido, Japan	Sea of Japan	42.32°N, 140.97°E	10	2.22 ± 0.07	1.22 ± 0.41	0.38
3. Esashi, Hokkaido, Japan	Sea of Japan	41.86°N, 140.18°E	25	3.41 ± 0.17	2.16 ± 0.68	0.52
4. Yazishi, Liaoning, China	Yellow‐Bohai Sea	39.04°N, 122.72°E	25	2.60 ± 0.13	0.00 ± 0.06	0.41
5. Dongbang, Liaoning, China	Yellow‐Bohai Sea	39.02°N, 122.75°E	25	2.96 ± 0.26	0.83 ± 0.76	0.37
6. Yingzuishi, Liaoning, China	Yellow‐Bohai Sea	39.01°N, 122.73°E	25	3.09 ± 0.13	1.42 ± 0.82	0.47
7. Lvshun, Liaoning, China	Yellow‐Bohai Sea	38.72°N, 121.15°E	24	2.83 ± 0.11	1.14 ± 0.57	0.39
8. Onagawa Bay, Miyagi, Japan	Japan‐Pacific coast	38.44°N, 141.48°E	18	2.88 ± 0.08	0.83 ± 0.38	0.30
9. Beihuangcheng Island, Yantai, China	Yellow‐Bohai Sea	38.38°N, 120.90°E	25	2.46 ± 0.07	0.00 ± 0.00	0.39
10. Daqin Island, Yantai, China	Yellow‐Bohai Sea	38.30°N, 120.83°E	25	2.81 ± 0.17	0.61 ± 0.56	0.39
11. Sokcho, Gangwon‐do, Korea	Sea of Japan	38.20°N, 128.59°E	14	2.08 ± 0.12	0.02 ± 0.14	0.28
12. Changdao Island, Yantai, China	Yellow‐Bohai Sea	37.97°N, 120.73°E	25	2.59 ± 0.11	0.23 ± 0.42	0.35
13. Jiming Island, Weihai, China	Yellow‐Bohai Sea	37.75°N, 122.80°E	25	3.00 ± 0.13	0.66 ± 0.52	0.49
14. Xiaoshi Island, Weihai, China	Yellow‐Bohai Sea	37.52°N, 122.01°E	24	2.10 ± 0.08	0.00 ± 0.00	0.32
15. Yueliang Bay, Weihai, China	Yellow‐Bohai Sea	37.51°N, 122.43°E	25	2.55 ± 0.09	0.00 ± 0.00	0.34
16. Chengshantou, Weihai, China	Yellow‐Bohai Sea	37.39°N, 122.71°E	25	2.63 ± 0.14	0.68 ± 0.59	0.40
17. Yantai University, Yantai, China	Yellow‐Bohai Sea	37.47°N, 121.46°E	25	2.33 ± 0.11	0.00 ± 0.00	0.24
18. Ailian Bay, Weihai, China	Yellow‐Bohai Sea	37.23°N, 122.59°E	25	2.65 ± 0.18	0.61 ± 0.49	0.43
19. Gauido, Chungcheongnam‐do, Korea	Yellow‐Bohai Sea	36.67°N, 126.07°E	6	1.81 ± 0.02	0.93 ± 0.25	0.32
20. Taean, Chungcheongnam‐do, Korea	Yellow‐Bohai Sea	36.66°N, 126.26°E	10	2.47 ± 0.09	0.37 ± 0.50	0.39
21. Badaguan, Qingdao, China	Yellow‐Bohai Sea	36.05°N, 120.35°E	25	2.57 ± 0.19	1.19 ± 1.00	0.34
22. Ama, Shimane, Japan	Sea of Japan	36.01°N, 132.58°E	11	3.40 ± 0.25	3.52 ± 0.87	0.52
23. Tateyama Bay, Chiba, Japan	Japan‐Pacific coast	35.00°N, 139.83°E	22	1.38 ± 0.09	0.87 ± 0.78	0.06
24. Chita, Aichi, Japan	Japan‐Pacific coast	34.71°N, 136.92°E	15	1.82 ± 0.00	2.61 ± 0.65	0.14
25. Yeongsan, Jeollanam‐do, Korea	Yellow‐Bohai Sea	34.65°N, 125.47°E	10	1.80 ± 0.21	0.00 ± 0.00	0.17
26. Jodo, Jeollanam‐do, Korea	Yellow‐Bohai Sea	34.32°N, 126.04°E	10	2.18 ± 0.12	0.98 ± 0.60	0.29
27. Jeju Island, Jejudo, Korea	Yellow‐Bohai Sea	33.28°N, 126.32°E	10	2.49 ± 0.29	1.33 ± 0.73	0.46
28. Shengsi, Zhoushan, China	East China Sea	30.71°N, 122.77°E	25	2.13 ± 0.45	0.02 ± 0.13	0.21
29. Dongtou, Wenzhou, China	East China Sea	27.80°N, 121.14°E	25	2.67 ± 0.14	0.00 ± 0.00	0.41
30. Longchuanjiao, Wenzhou, China	East China Sea	27.45°N, 121.05°E	25	2.55 ± 0.38	0.64 ± 0.53	0.30
31. Sanpanwei, Wenzhou, China	East China Sea	27.48°N, 121.05°E	25	1.88 ± 0.09	0.33 ± 0.47	0.26
32. Zhuyu Island, Wenzhou, China	East China Sea	27.46°N, 121.10°E	25	2.02 ± 0.18	1.22 ± 0.71	0.28
33. Huangqi, Liangjiang, Fuzhou, China	East China Sea	26.41°N, 119.92°E	24	2.21 ± 0.12	2.23 ± 0.64	0.25
34. Nanri Island, Putian, China	East China Sea	25.26°N, 119.67°E	29	1.97 ± 0.08	0.51 ± 0.50	0.27
35. Meizhou Island, Putian, China	East China Sea	25.07°N, 119.13°E	24	1.82 ± 0.07	1.86 ± 0.35	0.20

Eleven species‐specific polymorphic microsatellite loci were used to genotype all 35 populations (717 individuals) as detailed in a previous study (Li, Hu, Sun, et al., 2017). Among all genotyped localities, 22 populations from China were retrieved from Li, Hu, Sun, et al. (2017), and the remaining 13 populations from Korea and Japan were screened in this study. Allelic scores were checked manually for quality and consistency in STR_AND_ (Toonen & Hughes, 2001) using the 350 ROX™ size standard (Applied Biosystems).

GENEPOP 4.0 (Raymond & Rousset, 1995) was used to test linkage disequilibrium between each pair of microsatellite loci and deviations from Hardy–Weinberg equilibrium (HWE). MICRO‐CHECKER 2.2 (van Oosterhout et al., 2004) checked for null alleles in order to avoid bias in estimating genetic parameters. GENALEX 6.0 (Peakall & Smouse, 2006) calculated population‐level summary statistics of genetic diversity, including the number of alleles (*N*_a_), the effective number of alleles (*A*_e_), observed (*H*_o_) and expected heterozygosity (*H*_e_). FSTAT 2.9.3.2 (Goudet, 2002) determined the inbreeding coefficient (*F*_IS_), allelic richness ($\hat{\alpha }$) and private gene diversity ($\hat{\pi }$). Allelic richness and private alleles were standardized to the smallest sample size with 10^4^ randomizations. Pairwise *F*_ST_ and Jost's *D* were estimated with GENALEX. Genetic differentiation among 35 geographical populations was also explored by factor correspondence analysis (FCA) of multilocus scores using GENETIX (Belkhir et al., 2004).

The number of genetic clusters was inferred using STRUCTURE 2.3.4 (Pritchard et al., 2000) without *a priori* population assignment and allowing admixture. For each possible number of clusters (*K* = 1–10), the analysis ran with 5 independent simulations using a full‐length of 8×10^6^ iterations and a burn‐in of 10^6^ iterations, which were determined to be sufficient to obtain consistent results. The estimation of K used the log probability of the data Pr(*x*/*K*) and the Δ*K* criterion (Evanno et al., 2005). To compare genetic diversity and differentiation at the clustering level, $\hat{\alpha }$, $\hat{\pi }$, Jost's *D* and *F*_ST_ were also calculated in each genetic group identified by STRUCTURE analyses.

### 2.3 Species distribution modelling from past to future times

Species distribution models (SDM) were developed to test whether past climate‐driven range shifts explain the observed phylogeographical structure. The models were developed with the machine learning algorithm Boosted Regression Trees (BRT) using occurrence records against biologically meaningful environmental predictors for intertidal marine brown algae (Assis et al., 2017). These were nutrients (as nitrate and phosphate), salinity, sea ice thickness and sea surface temperature (maximum and minimum). Georeferenced occurrence data for the whole distribution of *S*. *thunbergii* were compiled from the Global Biodiversity Information Facility (GBIF), the Ocean Biogeographic Information System (OBIS) and scientific journal articles (Table S1). Environmental data for the surface of the ocean were downloaded from Bio‐Oracle (Assis, Tyberghein, et al., 2018). To reduce the negative effect of spatial autocorrelation (see Segurado et al., 2006), the correlation of environmental variables within the range of occurrence records was determined as a function of geographic distance with Mantel tests under 1 × 10^4^ permutations (e.g. Boavida et al., 2016).

The optimal parameters of BRT models (learning rate, number of trees and tree complexity; De'ath, 2007) were tuned by implementing a cross‐validation framework that partitioned occurrence records (both presences and pseudo‐absences) into 10 independent latitudinal bands (Assis et al., 2017). This approach aimed to reduce the effect of overfitting and increase the potential for temporal and spatial transferability. Performance was evaluated with the area under the curve (AUC) and sensitivity (true positive rate) (Araújo, & New, 2007; Assis et al., 2017).

The potential distributional range of *S*. *thunbergii* was hindcasted (LGM, MH) and forecasted (two contrasting emission scenarios RCP26 and RCP85) with information about phylogeographical structure (i.e. within‐taxon niche structure; Pearman et al., 2010). In this process, models were developed for each geographical region identified as genetically differentiated by dividing occurrence records into distinct datasets based on the identified genetic groups (e.g. Assis et al., 2016). Final predictions were reclassified to binomial responses (presence‐absence maps) with a threshold maximizing specificity (true negative rate, based on pseudo‐absences) and sensitivity (e.g. Assis et al., 2016, 2017).

To assess potential niche differences between the two genetic groups, we used a probabilistic method using a Bayesian framework to determine pairwise niche overlap (see Swanson et al., 2015 for details). An additional niche similarity method using 10^4^ randomizations tested whether the niche regions of the two genetic groups are more similar to each other than expected by chance (i.e. niche similarity, Broennimann et al., 2012).

### 2.4 ABC inference of demographic history

The obtained phylogeographical structure and SDM results further allowed us to test a few alternative hypotheses (see Introduction) concerning the evolutionary history of *S*. *thunbergii* in the Northwest Pacific. These analyses used demographical modelling and approximate Bayesian computation (ABC) as implemented in DIYABC 2.1.0 (Cornuet et al., 2014). The first hierarchical level of structural analysis revealed a south to north genetic break (see Results); we thus conducted a relevant ABC analysis to test how these two groups established the current distribution pattern through glacial survival and postglacial colonization. We tested three broad‐scale vicariance hypotheses: (i) *S*. *thunbergii* survived in a single southern refugium and expanded northward postglacially; (ii) it survived in a single northern refugium during glaciation and expanded southward postglacially; (iii) the northern and southern groups survived as separated glacial relics, respectively, from an extinct common ancestor. The divergence between the two groups could have occurred before or after the LGM, thus generating six competitive scenarios (Figure S1). For each scenario, the current populations were set to time t_0_, and the divergence from the most recent common ancestor (MRCA) was set to t_1_ (post‐LGM) or t_2_ (pre‐LGM). The mutation rate of microsatellites (10^−4^–10^−3^) was inferred from the phylogenetically close species *Fucus* (Pereyra et al., 2009). The prior values of effective population size for the southern and northern groups are listed in Table S2.

The ABC analysis generated 10^6^ simulations for each scenario. The relative posterior probability of scenarios was estimated using a weighted polytomous logistic regression method on the 1% of the simulated data sets closest to the observed data sets (Cornuet et al., 2014). Taking into account the potential lack of confidence in ABC model selection (Robert et al., 2011), we calculated the posterior predictive error over 1000 data sets to empirically evaluate the power of the model to discriminate among scenarios. In addition, we chose the mean number of alleles, mean gene diversity and mean allele size variance across loci for single sample statistics as well as the *F*_ST_ values, mean index of classification and (*δµ*)^2^ distance for two sample statistics.

## 3 RESULTS

### 3.1 Genetic diversity patterns and lineage structure

No evidence of null alleles nor linkage disequilibrium was found between the 11 microsatellite loci. Consequently, the 11 selected microsatellite loci yielded 111 alleles (6–18 per locus). The mean percentage of polymorphic loci in all 35 populations was 86.75%, with the lowest value (27.27%) found in the samples from Tateyama Bay and Chita, Japan. The samples from Esashi, Hokkaido and Ama, Shimane, Japan exhibited the largest number of alleles (*N*_a_ = 3.64/3.73, *A*_e_ = 2.28/2.36). Gene diversity (*H*_e_) ranged from 0.06 in Tateyama Bay, Chiba, Japan to 0.52 in Esashi, Hokkaido and Ama, Shimane, Japan, respectively (Table 1). Allelic richness showed a similar pattern to *H*_e_ across latitudes, whereas private alleles were most frequent ($\hat{\pi }$ = 3.52) in Ama, Shimane, Japan relative to other localities ($\hat{\pi }$ ≤ 2.61). The Yellow‐Bohai Sea samples, particularly 9–12, 14, 16 and 18–27, exhibited relatively fewer private alleles (Table 1). *H*_o_ did not significantly differ from *H*_e_. Mean estimates of *N*_a_, *A*_e_, *H*_o_ and *H*_e_ over loci and samples were 2.623, 1.727, 0.334 and 0.342, respectively. HWE tests revealed little evidence for significant deviations from equilibrium expectations. FSTAT analyses showed that few (6/35) inbreeding coefficients (*F*_IS_) were significantly positive.

All *F*_ST_ and Jost's *D* estimates were significantly differentiated (*p* < 0.01). *F*_ST_ and Jost's *D* revealed lower genetic differentiation within the northern and southern limits (north: mean *F*_ST_ 0.195, mean Jost's *D* 0.362; south: mean *F*_ST_ 0.132, mean Jost's *D* 0.116; Tables S3 and S4), and higher differentiation within the core populations (north: mean *F*_ST_ 0.331, mean Jost's *D* 0.487; Tables S3 and S4).

**FIGURE 2:**
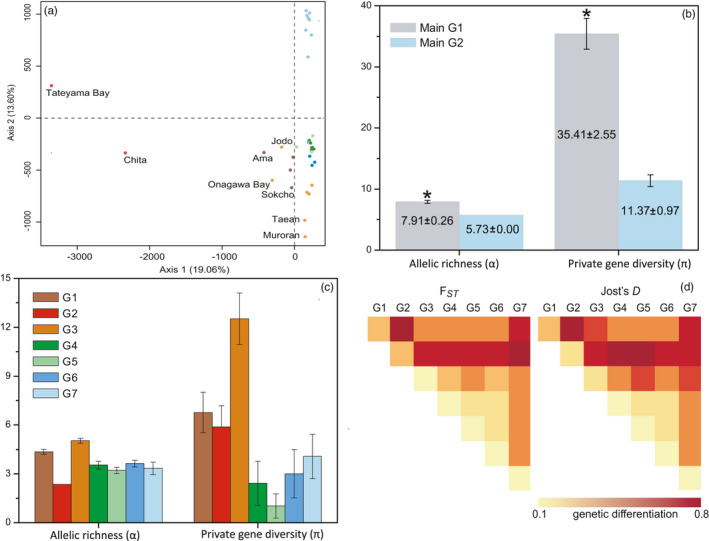
(a) Genetic differentiation inferred by factorial correspondence analysis (FCA) of population multiscores. Standardized allelic richness and private diversity (number of unique alleles) for the first (b) and second (c) hierarchical level of genetic subdivisions. Colours refer to the same assignment of genetic clusters in Figure 1. Asterisks refer to significant higher levels of diversity. (d) The genetic differentiation matrix of average *F*_ST_ and Jost's *D* between 7 subdivided clusters

Structure analysis revealed two major levels of genetic subdivision in *S*. *thunbergii* in the Northwest Pacific. The first hierarchical level divided a southern genetic cluster comprising all 8 populations in the southern limits from the core‐northern limit cluster (Figure 1a). The second level (the optimal value of Δ*K*, Figure S2) divided the core‐northern cluster into 6 sub‐clusters of which 3 were endemic to the Yellow‐Bohai Sea, and the remaining occurred along the Korean and Japanese coastlines (Figure 1b,c). Genetic subdivisions of K set from 3 to 6 are presented as Supporting Information (Figure S3). FCA also revealed striking genetic subdivisions in *S*. *thunbergii*, with the first two axes accounting for 32.66% of the variation observed. The most distant (first axis, 19.06% of the variation) genetic group is that comprised by Tateyam Bay and Chita, from the Japan‐Pacific coast, which were clearly highly differentiated from all other populations and also among themselves (Figure 2a/d, Tables S4, S5). The second axis (13.60%) separated samples from the southern range cluster from those in the core‐northern limit (Figure 2a), in agreement with the first separation in the STRUCTURE analysis which underlines Hardy‐Weinberg expectations rather than population distances.

At the first hierarchical level, allelic richness was lower (5.73) in the southern than the core‐northern range (7.91), and the number of private alleles in the core‐northern limit (35.41) was over 3‐fold larger than in the southern cluster (11.37) (Figure 2b). Allelic richness per genetic cluster (second hierarchical level) was significantly greater in G1, which comprised Shakotan, Esashi, Ama, Japan and Jeju Island, Korea than in G3, which consisted of Muroran and Onagawa Bay, Japan, and in 5 localities in Korea. Much more private diversity ($\hat{\pi }$ = 12.53) was found in G3, which was as rich as *c*. 2‐fold higher than in G1 and G2 and *c*. 3‐ to 5‐fold larger than in G4, G6 and G7 (the southern limit) (Figure 2c). The Ama subpopulation made an important contribution to the endemism in G3, as the number of private alleles dropped to 7.16, excluding Ama. *F*_ST_ and Jost's *D* indicated that the 7 genetic clusters were significantly differentiated from each other, particularly G2, which included the highly diverged Tateyama and Chita (Figure 2d). The curves derived from the linear regression of *N*_a_, *A*_e_, *H*_o_ and *H*_e_ over latitudes consistently indicated a slow but distinct decline from the northern limit (>39°N) to the centre (39°N < 28°N), and to the southern limit (<28°N) of the species range (Figure S4). No isolation‐by‐distance pattern was found in the entire distribution range of *S*. *thunbergii* (Figure S5), because *P*‐value was statistically nonsignificant (data not shown).

Differentiation among populations within genetic clusters was greater in the range centre, followed by the northern and southern range limits, respectively (Tables S4, S5).

### 3.2 Range shifts of *Sargassum thunbergii* from past to future

From 231 initial records of occurrence compiled from the literature and databases, a final set of 81 records resulted from considering the minimal noncorrelated distance of 15 km (Figure S6). The BRT algorithm shows that the distribution of the species in the northern region was mostly explained by sea ice thickness (72.53%) and maximum ocean temperatures (MaxOT, 26.17%), and in the southern region by minimum ocean temperature (32.30%), followed by MaxOT (31.88%), phosphates (17.14%) and nitrates (14.48%) (Figures S7, S8, Table S5). The northern and southern groups in *S*. *thunbergii* have different inferred tolerance limits to salinity and nitrates (Table 2). The cross‐validation had high accuracy scores (Northern: AUC = 0.87 ± 0.12, sensitivity = 0.83 ± 0.26; Southern: AUC = 0.94 ± 0.10, sensitivity = 0.83 ± 0.41). For both the northern and southern groups, the final prediction performed by ensemble modelling also retrieved high discriminatory power (AUC = 0.97, sensitivity = 0.97) (Table 2).

**TABLE 2:** Performance (i.e. accuracy scores) of boosted regression trees considering within‐taxon niche structure (northern and southern genetic groups, as well as the ensemble of both models)

Grouping	Cross‐validation		Final prediction	
	AUC	Sensitivity	AUC	Sensitivity
Northern group	0.8740 ± 0.1199	0.8333 ± 0.2582	0.9759615	1
Southern group	0.9375 ± 0.1046	0.8333 ± 0.4082	0.9354167	0.9375
Ensemble	‐	‐	0.9722317	0.9736842

**FIGURE 3:**
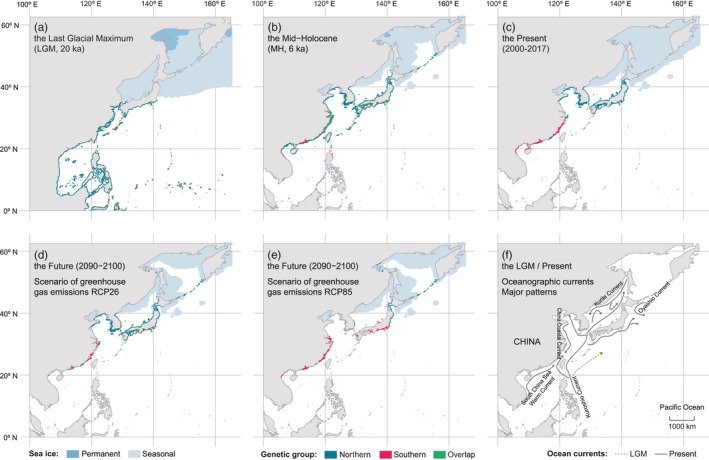
Ensemble distribution maps of *Sargassum thunbergii* for the Last Glacial Maximum (LGM) (a), the mid‐Holocene (MH) (b), the present (c) and the future (2090–2100) (d and e), and major oceanographic currents during the LGM and present‐day conditions (f) (drawn after Hu et al., 2011). The future distribution considered contrasting scenarios of greenhouse gas emissions of RCP26 (d) and RCP85 (e). Predictions were performed with the ensemble of models considering genetic structure. Light blue polygons depict seasonal sea ice and grey polygons depict land area

Hindcasting to the LGM revealed a more southern distribution of *S*. *thunbergii* than today, with the southernmost limit extending to North Sulawesi, Indonesia (*c*. 0°N). Suitable habitats were available along the coast of China and northward reached the north of the Pacific coast of Japan (*c*. 38°N) (Figure 3a). From the LGM to the MH, SDM predicted a profound reshaping of the distribution, with a *c*. 18 degrees of latitude of contraction in the southern edge to Hainan Island, China and northerly range‐wide (*c*. 12°N) expansion to Severo‐Kurilsky, Russia (*c*. 50°N) (Figure 3b). Comparing the predicted distributions for the past and present times also revealed interesting features of past range shifts involving the lack of habitats in the Sea of Japan (due to sea ice thickness during the LGM) and in the Yellow and Bohai Seas (due to sea‐level variation during the LGM) (Figure 3a,b).

SDM predictions for the present‐day matched the present known distribution of *S*. *thunbergii*. They indicated that the northern group was mainly restricted to the Japanese archipelago, the Korean peninsula and the Yellow‐Bohai Sea, whereas the southern group predominantly occurred in East China Sea (Figure S9). Compared to the MH, there were 8 degrees of latitude of range contraction in the north whereas the southern range expanded northward considerably (Figure 3c). Comparing the predicted distributions for the future (Figure 3d,e) allowed verifying that suitable habitats would not to be completely extinct in the southern range (as predicted for most species across the globe). Increasing greenhouse emissions are predicted to produce massive northern expansions along the Kuril Islands and the extinction of the South Korean peninsula populations (Figure 3d,e).

The posterior distribution of the probabilistic niche overlap metric between the northern and southern genetic groups is 37.3%, while the overlap between the southern and northern groups is 38.1% (Figure S10). Niche similarity test retrieved a similar overlap of 39%, with a *p*‐value of 0.01; rejecting the null hypothesis of climatic niches being more similar to each other than expected by chance.

### 3.3 Demographic history in a model‐based framework

Model‐based inference at the first hierarchical level pointed to the southern group having persisted during the LGM, from which the northern group was derived through stepping stone colonization (Scenario 3; Figure S1). The southern source scenario during the LGM was supported with the maximum posterior probability (0.9696, 95% CI: 0.9642–0.9749) relative to almost zero support for the other five scenarios, including colonization from the northern source and for each deriving from an extinct common ancestor separately (Figure S1). The estimated divergence between the southern and northern groups occurred *c*. 4790 generations ago (95% CI: 1400–11200), corresponding to a timeframe around 5–10 ka (95% CI: 1.4–22.4 ka, i.e. between the end of the LGM and the Holocene). The evaluation confidence in scenario 3 showed that the posterior predictive error computed over 1000 datasets was 0.058, indicating a reliable model selection.

## 4 DISCUSSION

This study combining empirical genetic data with independent theoretical modelling shows how climate‐driven distributional shifts in the Northwest Pacific structured present *S*. *thunbergii* gene pools and threaten their future. Populations that persisted in refugial regions during the LGM produced divergent northward expansions (18 degrees of latitude) and southern range contractions, with the risk of erosion of the current levels of genetic diversity. Projecting the distribution of *S*. *thunbergii* populations under contrasting scenarios of future climate change further showed unique gene pools in imminent risk of disappearing.

### 4.1 Latitude‐wide genetic diversity patterns in coastal marine floras

The theory predicts that leading and rear edges of a species distribution are expected to have low genetic diversity, which at the rear edge might be low within populations but with high genetic differentiation among populations due to high genetic drift, geographical isolation and selection (Eckert et al., 2008). However, this pattern was not verified in *S*. *thunbergii*. Rather, cryptic refugia, long‐distance dispersal and postglacial expansion seem to have created a complex pattern of distribution range shifts and genetic diversity. Such processes are common in seaweeds (Assis et al., 2017; Assis, Serrão, et al., 2018; Hu et al., 2011; Li, Hu, Gao, et al., 2017; Neiva et al., 2015), but were not yet understood for the complex shorelines of the Northwest Pacific.

In the Northeast Atlantic, the leading and rear edge populations of coastal floras along the same coastline exhibit contrasting genetic diversity patterns that reflect past history rather than present habitat gradients. For example, the seagrass *Zostera marina* is characterized by lower genetic diversity at the northern and southern range limits, and richer diversity hotspots in the centre of its distribution (Diekmann & Serrão, 2012), yet, the underlying drivers of diversity are distinct in the northern expanding/leading edge (recent founder events) versus the southern rear edge (drift and bottlenecks), where habitat suitability is increasingly threatened. However, no geographical pattern was assigned when the southern range populations of *Zostera marina* were grouped as a whole, because different populations lose alleles independently, while globally they still retain high ancient genetic diversity (Diekmann & Serrão, 2012). Such processes were also detected in several other fucoid and kelp species, for which both genetic diversity within populations and differentiation among populations decrease northward from a southern range hotspot that still retains maximal diversity despite recent local bottlenecks, lower population density and higher habitat fragmentation (Assis, Serrão, et al., 2018; Neiva et al., 2015). These studies stress that genetic variation patterns of coastal floras at the southern range limit can be structured by both contemporary (e.g. range dynamics and dispersal) and historical events (e.g. population bottlenecks, extinctions, colonizations and survival during ice ages) (Hampe & Petit, 2005).

Our results showed that *S*. *thunbergii* populations at the southern range limit retain less diversity (Figure S4) and, despite being genetically isolated from more northern populations, the differentiation levels among populations within this region was the lowest (Tables S4, S5). Diversity was higher in the northern edge limit and central region, as expected considering that they comprise multiple genetic groups. Such pattern of genetic structure differs considerably from recent observations in the Northeast Atlantic (Assis, Serrão, et al., 2018; Diekmann & Serrão, 2012; Neiva et al., 2015).

The unique genetic variation in *S*. *thunbergii* likely stemmed from glacial survival in the southern range, with subsequent postglacial colonization northward. This key event of the species diversification was inferred to occur in the postglacial period of 5–10 ka (ABC analysis). While significant global sea‐level rise had an earlier start at ~14.5 ka (Clark et al., 2009), only at ~10 ka did the coastal morphology and the patterns of sea surface temperatures resemble those of present‐day conditions (Shintani et al., 2008; Zheng et al., 2016). In fact, the region comprising the southern range *S*. *thunbergii* had a slower warming rate during the last deglaciation, when compared to other regions of the world (Shintani et al., 2008). At the same time, selective adaptation to local environments (e.g. the northward‐flowing warm Kuroshio branch current and the southward flowing cold Oyashio current along the Japan‐Pacific coast) could have potentially contributed to mutation accumulation and hence fine‐scale diverged lineages of *S*. *thunbergii* in the northern range.

### 4.2 Glacial persistence and post‐LGM range shifts shaped genetic uniqueness

The low sea levels during the LGM devastated most near‐shore habitats in the Northwest Pacific (e.g. the Yellow‐Bohai Sea) and imposed drastic range shifts in the extant coastal species along their geographical ranges (Wang, 1999). Generally, populations persisted locally in glacial refugia or were recently colonized from periglacial areas (Provan & Bennett, 2008). In this study, two hierarchical analyses consistently divided *S*. *thunbergii* populations south of 31°N into a unique genetic cluster (Figure 1). This cluster, along with its relatively low levels of genetic diversity at both the population and clustering levels (Figure 2, Table 1), suggests the effect of a postglacial expansion from a presently extinct southern glacial refugium (e.g. the Hainan Island), or bottlenecks and drift owing to population reductions exposed to extreme conditions (Hu et al., 2018; Provan & Bennett, 2008).

Surprisingly, the Yellow‐Bohai Sea harboured three genetic clusters (G4–G6) in the second hierarchical analysis (Figure 2c,d). The intertidal to lower supratidal distribution of *S*. *thunbergii* implies that it might not have persisted the harsher environments (e.g. ice scouring and freezing) of the Bohai Sea during the LGM, but suitable habitats might still have been available in the Yellow Sea and the west Pacific (e.g. the Ryukyu archipelago) (Figure 3a). The clusters G4–G6 likely arose from post‐LGM expansions from glacial and/or periglacial ancestral relics in some areas, such as Jeju Island (Lee et al., 2012) and/or the Okinawa Trough (Hu et al., 2011, 2015), driven by the Kuroshio current system (Li, Hu, Gao, et al., 2017). The subsequent genetic erosion and founder effects might have left the pattern of relatively few private alleles in the Yellow‐Bohai Sea.

The genetic richness in each of the subdivided groups G1–G3 (Figure 2c) seems also to be derived from the survival of *S*. *thunbergii* in glacial refugia during the LGM, as reported in the Northwest Pacific (e.g. *Chondrus ocellatus*, Hu et al., 2015; *Sargassum fusiforme*, Hu et al., 2017) and the Northeast Atlantic (e.g. *Palmaria palmata* and *Fucus serratus*, Maggs et al., 2008). But this presumption was rejected by ABC analysis (Figure S1), which instead showed that the northern group, including G1‐G3, derived from the post‐LGM northward colonization of the southern group. Although the lumping of all of the northern populations together could be affecting the inference, the population size in the northern group is significantly larger than the southern (Table S1), which contrasts with the prediction that expanding populations should have low effective population size due to high genetic drift. Despite the short evolutionary time since the post‐LGM expansion, potential adaptation to novel environments (habitat quality and stability) in the centre‐northern range limit of *S*. *thunbergii* (Braasch et al., 2019) could drive rapid evolutionary sweeps, but the genetic markers used in this study are not expected to be affected by selection. The most plausible explanation to the observed patterns of distinct genetic lineages is supported by the unique alleles in the southern group coupled with the potential genetic loss in such source population, a hypothesis that is in agreement with our modelling results of past distributional shifts. The estimated divergence timing coincides with the postglacial range expansion rather than with allopatric isolation during glacial periods, suggesting that genetic divergence is caused by the population genetic bottleneck consequences of demographic processes linked to prior colonization by the genotypes present at the expanding margins.

SDM predicted contrasting distribution patterns between historical and present‐day populations. During the LGM, the drastic drop of sea‐level and environmental changes caused a substantial contraction of niche area for *S*. *thunbergii*. This process led to a southward range‐wide shift and colonization of the South China Sea and North Sulawesi, Indonesia, including the Philippine Archipelago (Figure 3a). Such a model hindcast might have not overestimated such distribution during the LGM considering that (i) the sea surface temperatures in the South China Sea were 8–13°C during the LGM (Zhou et al., 2007) and did not exceed the current growth and survival temperature limits of *S*. *thunbergii* (4–29°C in Maizuru Bay, Japan (Umezaki, 1974); 4.2–25.8°C at Padori, Korea (Koh et al., 1993)); (ii) previous studies showed that this cold‐temperate species can occur along the entire coast of China, including the southernmost Guangxi and Hainan provinces (Titlyanova et al., 2014; Tseng, 1983). During the MH, the distributional range of *S*. *thunbergii* was predicted to shift northward into the Sea of Japan and the Pacific coast of northern Japan where rising sea levels brought in the warm Kuroshio Current (Park & Park, 2015). The predicted pattern of southern decline and northern expansion of niche suitability from the LGM to the present could have also occurred in additional benthic species along the Northwest Pacific coastlines (Polovina et al., 2011).

### 4.3 Conserving unique gene pools under climate warming

Genetic diversity patterns often reflect historical extinctions, dispersal and colonization events, rather than processes resulting from current ecological conditions. Inferring past distributional shifts and genetic diversity levels can provide the requisite information for contemporary conservation and management (Morelli et al., 2016), particularly for coastal floras that are vulnerable to marine climate change (Wernberg et al., 2018). In this study, the predominant role of range‐wide shifts from the LGM to the present in shaping the genetic structure and diversity of *S*. *thunbergii* highlights the importance of niche conservation under climate change. This is particularly important for species like *S*. *thunbergii* that survived past climate extremes in habitats that remained suitable in the long‐term, but are currently in risk of disappearing, and with it so will those unique genetic pools, as species shift distributions poleward (Lavergne et al., 2013).

The IPCC reported that the global temperature has warmed 0.85°C over the past 130 years and will be at least 1.5°C higher than in the preindustrial era by the end of 21st Century (IPCC, 2013). Recent predictions showed that the coastal SSTs of the West, Yellow, East and South China Seas have been warming up to 0.5°C per decade from 1982 to 2010 (Lima & Wethey, 2012). Global warming can cause local population extinctions and the loss of unique genetic diversity in brown seaweeds (e.g. east Atlantic; Lourenco et al., 2016). Our year‐round field surveys indicate that the southern rear edge of *S*. *thunbergii* (e.g. Guangxi and Guangdong provinces, China) have already experienced range‐wide distribution losses since the 1980 s (Tseng, 1983). Our future predictions under warmer climate scenarios anticipate the southern range limit of *S*. *thunbergii* populations (e.g. East China Sea) even further isolated from the core‐northern limit, owing to fragmented habitats and environmental pressures, particularly under the greenhouse gas emission scenario of RCP85 (Figure 3e).

These changes caused by warming can be magnified by superimposed local stressors that might act synergistically in deteriorating the future habitat conditions for the species. Alongside thermal conditions, the SDM models further underlined the role of nutrient and salinity regimes in explaining the southern range edges (Table S5). Continental runoffs with high nutrient concentrations from agricultural areas are already a documented concern along the coastlines of the China Sea (Breitburg et al., 2018), which can reduce habitat suitability due to local eutrophication. The additional impact of such stressors in the future distribution of *S*. *thunbergii* can be further magnified owing to the projected precipitation regimes for the region, which are suggested to increase both in frequency and intensity (Guo et al., 2018). The increase in heavy rains might further impact negatively future habitat conditions, particularly if salinity levels drop below physiological tolerance limits for any life stage, such as the most vulnerable reproductive cells.

The ongoing range shifts create multiple challenges for conservation and resource management (Lima & Wethey, 2012). The northern range shifts may first allow *S*. *thunbergii* to colonize the colder high‐latitude environments to the north of Hokkaido, Japan, producing unpredictable effects on the recruitment, structure and interaction of native coastal marine species in these ecosystems (Bertness et al., 1999). Also, the unique genetic richness of *S*. *thunbergii* is under risk of erosion and loss in the northern periphery, including the Japanese Archipelago and Korean Peninsula, due to current and future climate changes. For example, the southwest of Japan witnessed a large increase of 0.3°C per decade in the annual mean SST since the 1970 s, which caused a successive contraction of distribution range of three cold‐temperate *Sargassum* species (*S*. *okamurae*, *S*. *micracanthum* and *S*. *yamamotoi*) (Tanaka et al., 2012). Thus, real‐time and long‐term monitoring of range shifts of biomass and distribution of *S*. *thunbergii* in the northern range may provide additional information to implement conservation plans, as our results provide guidance for consideration of gene pools in management under climate changes.

## CONFLICT OF INTEREST

None declared.

## AUTHOR CONTRIBUTIONS

Z.‐M.H., J.A. and E.A.S. conceived the project. Z.‐M.H., X.‐H. S., X.G. and H.‐G.C designed sampling and collected specimens, X.‐H. S. and J.Z. conducted molecular experiments, X.‐H. S., J.A. and J.Z. analysed molecular data, Z.‐M.H., X.‐H. S., J.A., E.A.S. and D.‐L.D. interpreted the data and wrote the manuscript. All authors approved the final version of the manuscript.

## Supporting information

Supplementary Material

## Data Availability

Molecular data sets, genetic estimates and Species Distribution Modelling input files are available at Dryad Digital Repository: https://doi.org/10.5061/dryad.0gb5mkkwm to be completed after manuscript is accepted for publication.
