# Supplementary material for: Climate‐induced range shifts shaped the present and threaten the future genetic variability of a marine brown alga in the Northwest Pacific
Source: Evol Appl. 2021 May 18;14(7):1867–79. doi: 10.1111/eva.13247 (PMC8288013; doi:10.1111/eva.13247)
Supplement: Supplementary file 1 — Supplementary Material [file EVA-14-1867-s001.pdf]

Supplemental Information

Climate-induced range shifts shaped the present and threaten the future genetic variability of a marine brown alga in the Northwest Pacific

Table of Contents:

Figure S1	Page 2
Figure S2	Page 3
Figure S3	Page 4
Figure S4	Page 5
Figure S5	Page 6
Figure S6	Page 7
Figure S7	Page 8
Figure S8	Page 9
Figure S9	Page 10
Figure S10	Page 11
Table S1	Pages 12-13
Table S2	Pages 14
Table S3	Pages 15-16
Table S4	Pages 17-18
Table S5	Page 19

Supplemental Figures

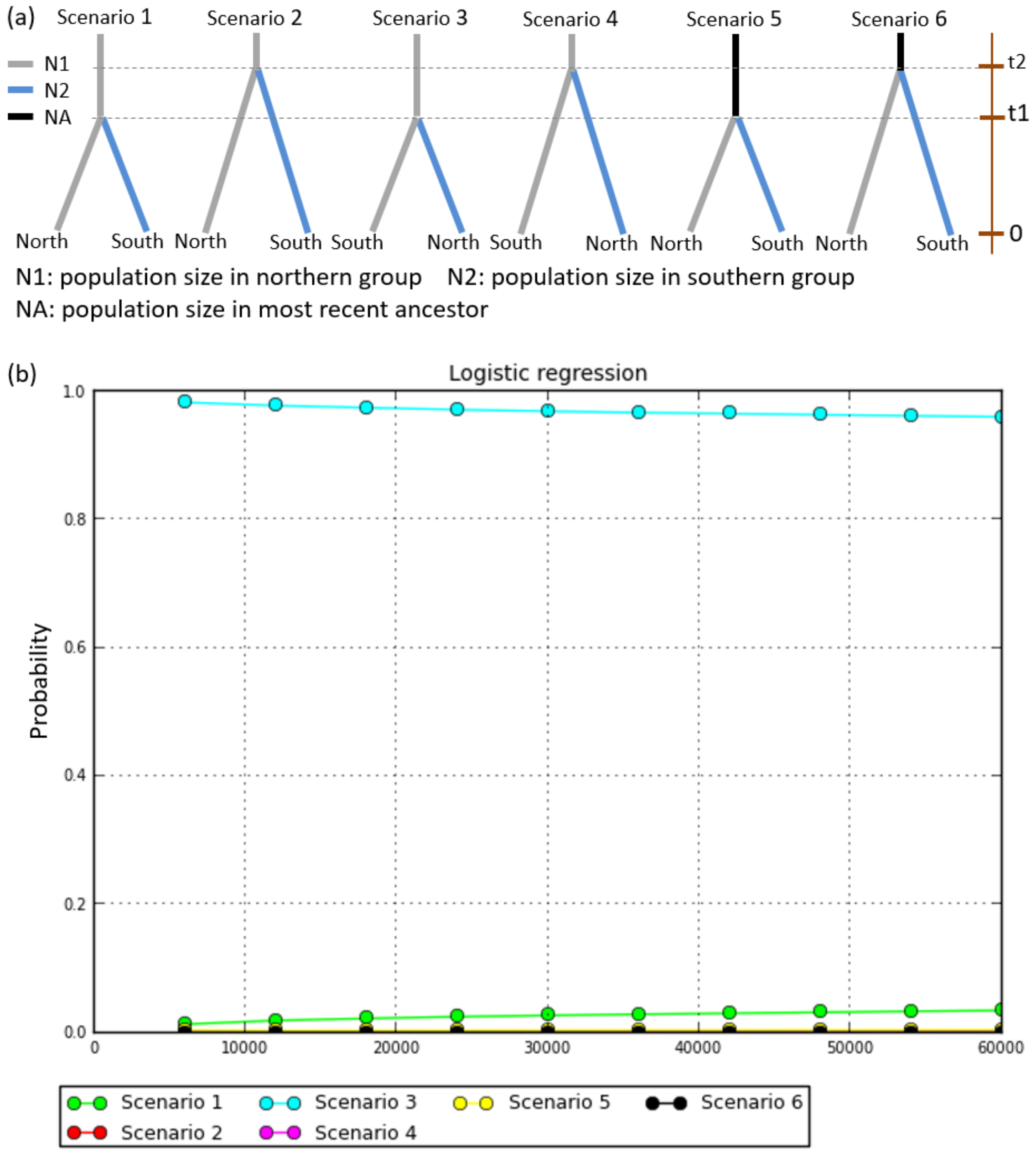

Figure S1. Schematic representation of scenarios compared in the first hierarchal levels of ABC analysis. The southern edge of the range (southern lineage) can be derived from stepping stone colonization of the northern lineage after the LGM (scenario 1) or before the LGM (scenario 2); the northern lineage can be derived from the stepping stone colonization of the southern edge of the range after the LGM (scenario 3) or before the LGM (scenario 4); the northern and southern lineages derived from an extinct most common ancestor and evolved separately after the LGM (scenario 5) or before the LGM (scenario 6) . The logistic regression of the posterior probabilities of the three scenarios (b) was presented as a function of number of simulated datasets.

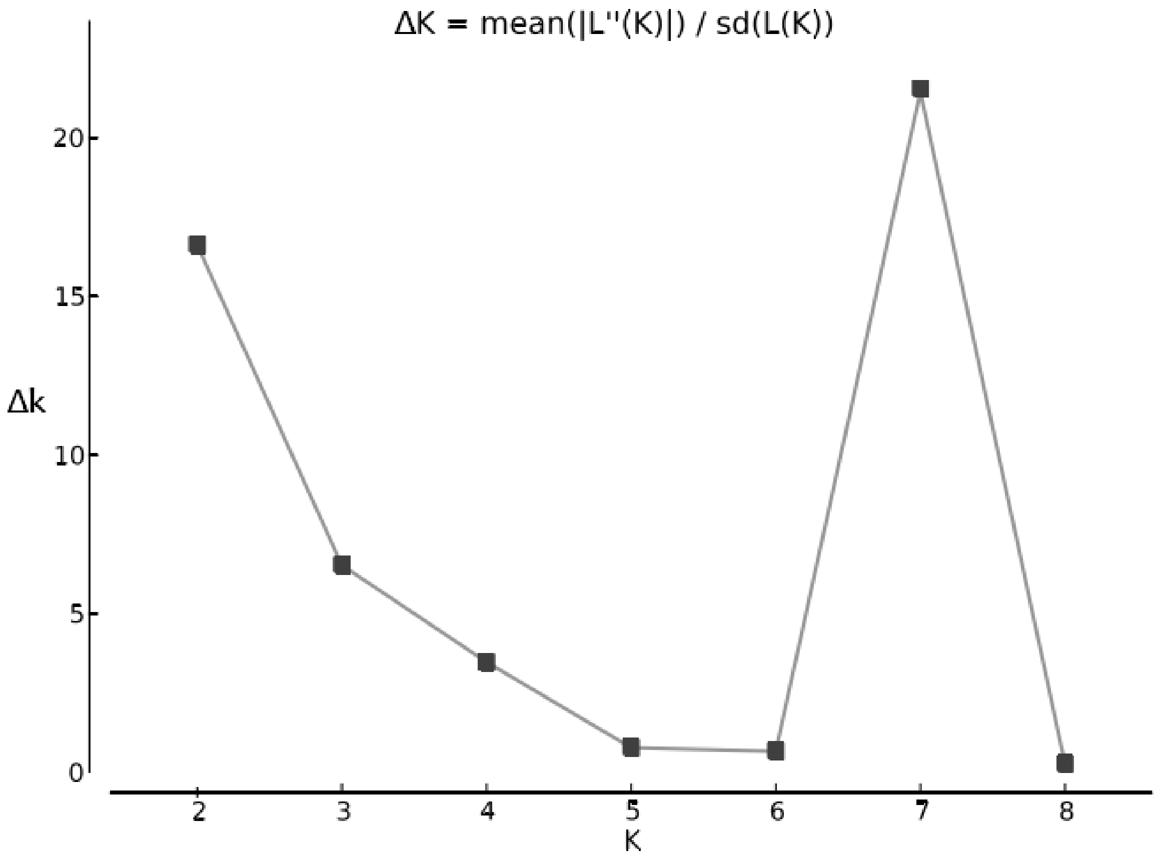

Figure S2. Plot of ΔK calculated as the mean of the second-order rate of change in likelihood of K divided by the standard deviation of the likelihood of K.

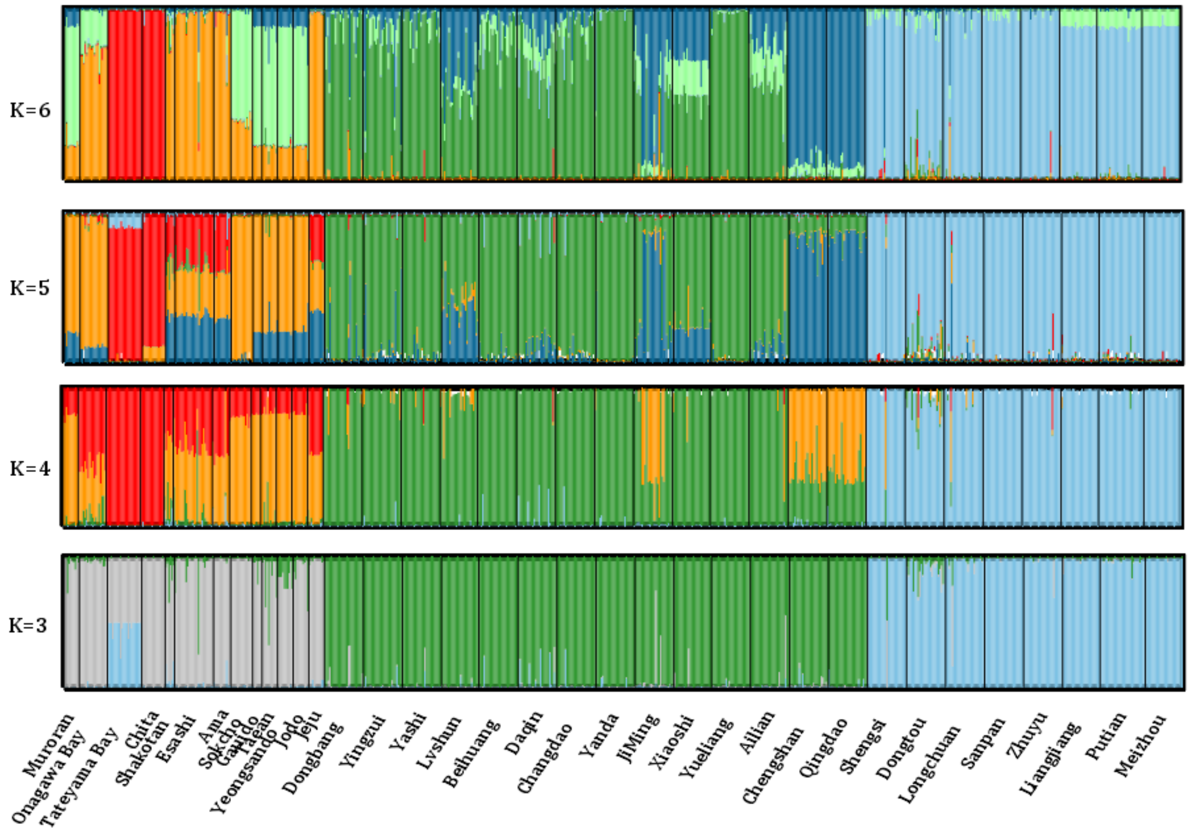

Figure S3. Genetic subdivision in *Sargassum thunbergii* based on Structure grouping (k=3-6). Colors are the same as depicted in Figure 1.

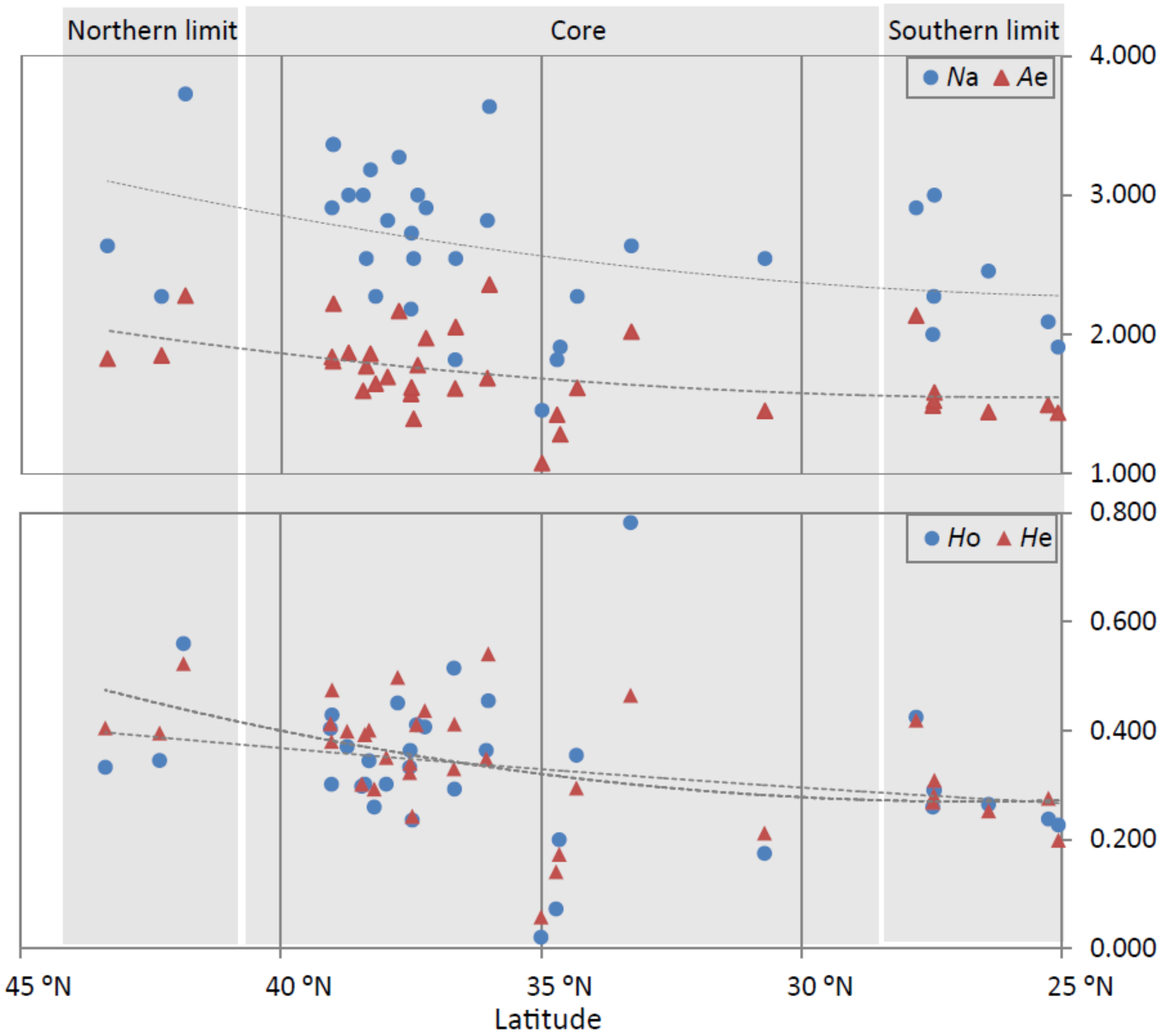

Figure S4. Latitudinal gradient genetic diversity measures (N_a, A_e, H_o, H_e) from the northern limit to the core and to the southern limit of the species' range.

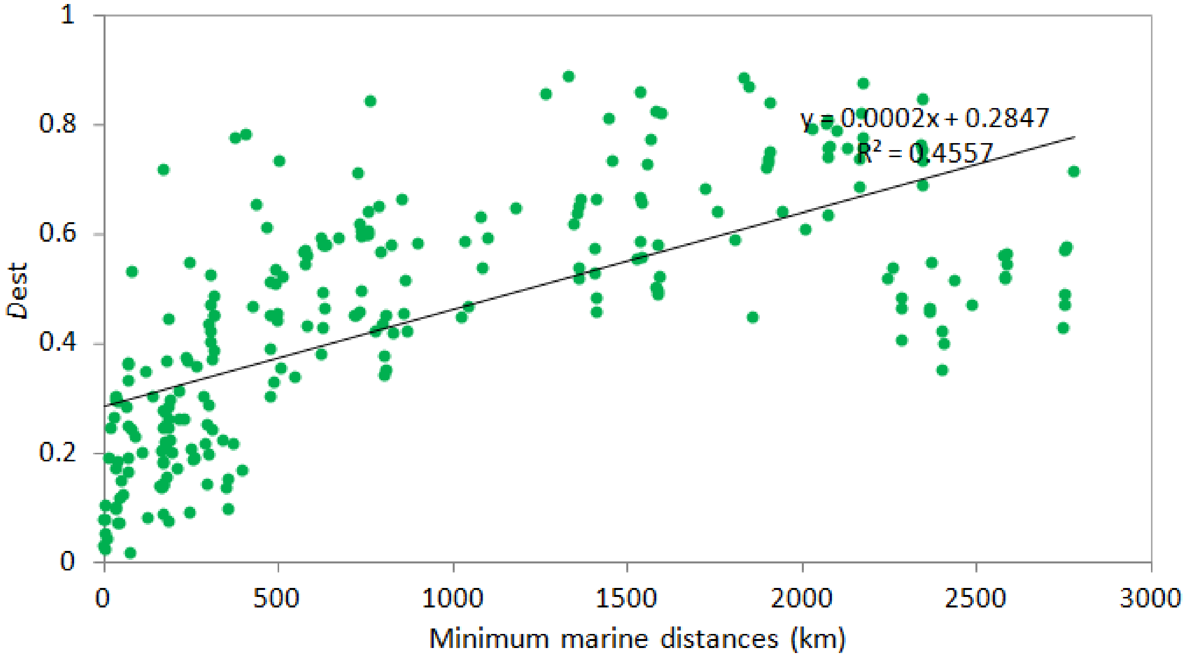

Figure S5. Estimates of pairwise differentiation (D_{est}) in *Sargassum thunbergii* plotted against minimum marine distance for the range of the species. Isolation by distance was non-significant across the entire range.

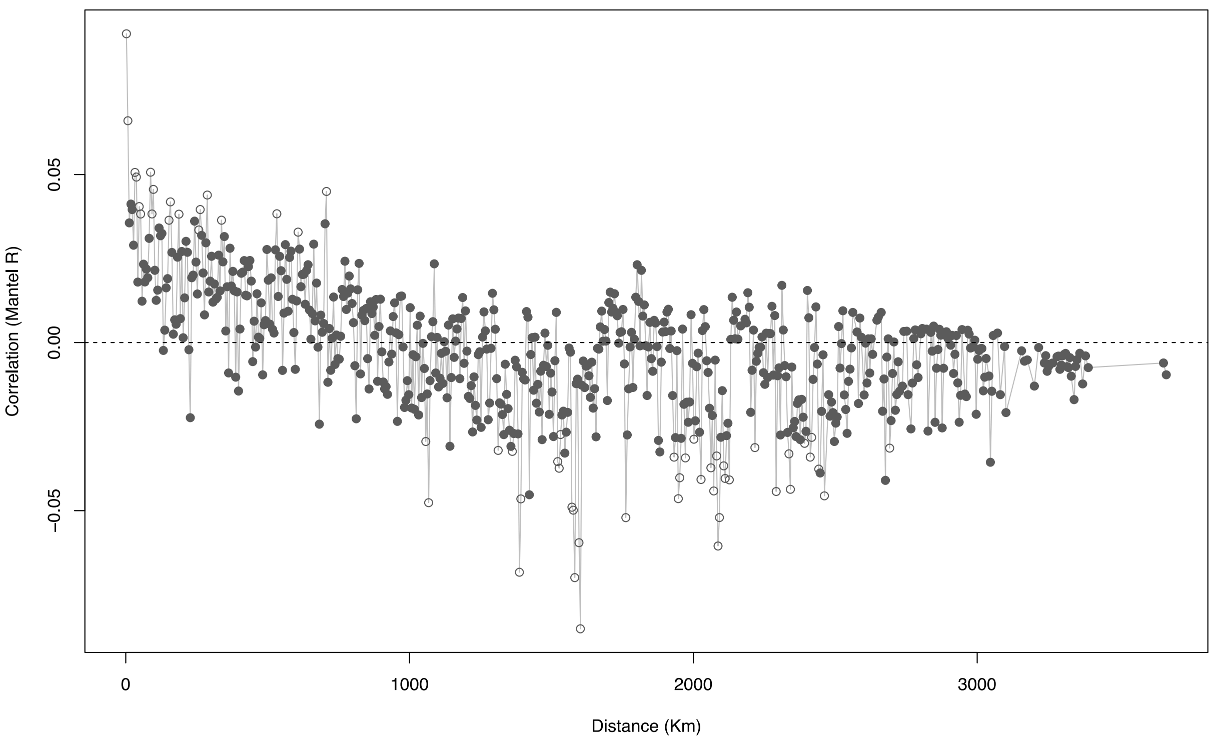

Figure S6. Spatial autocorrelation of environmental predictors (open circles depict significant spatial correlation).

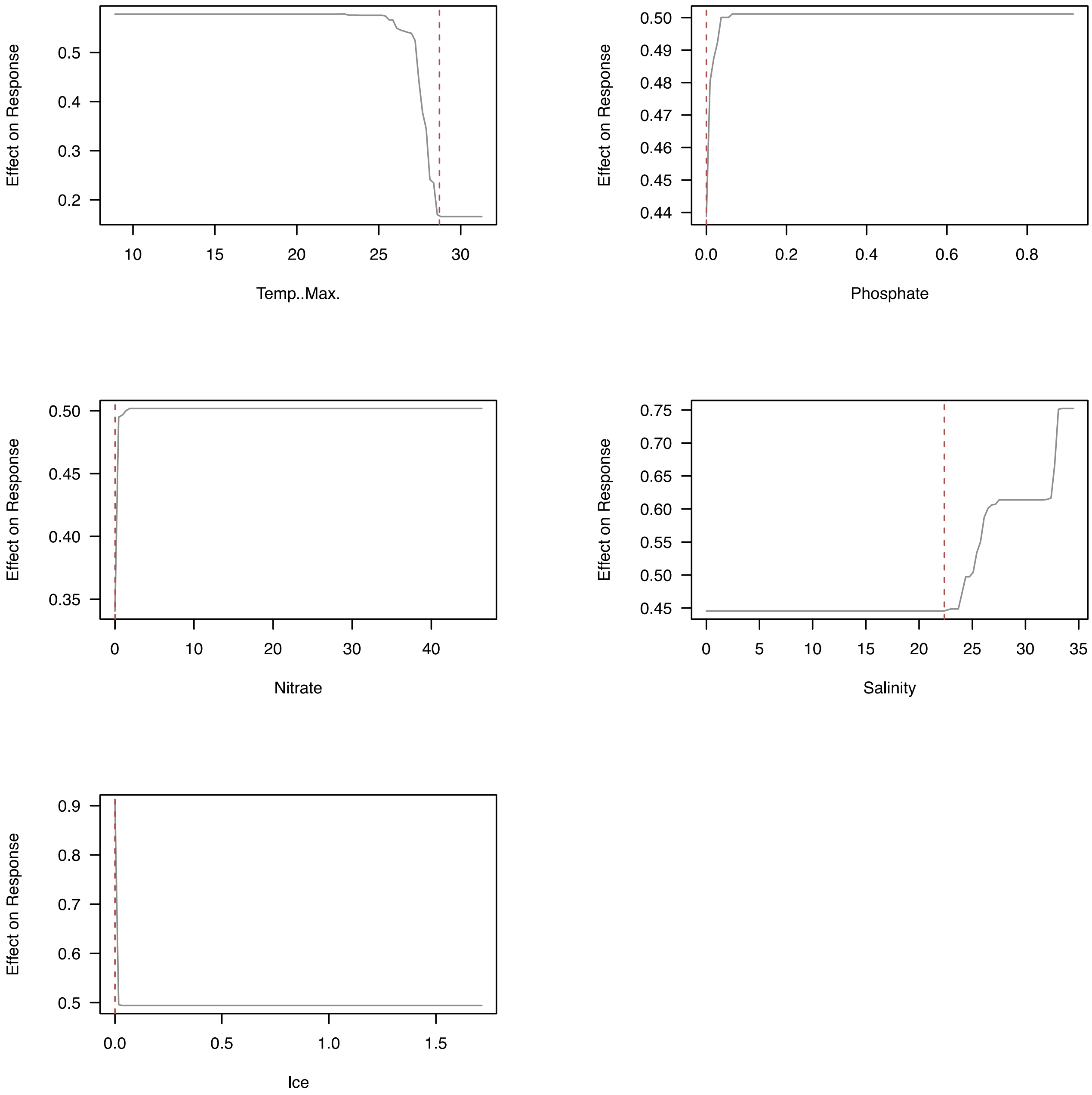

Figure S7. Partial dependency plots depicting the effect of each single predictor to the models developed for the northern genetic group.

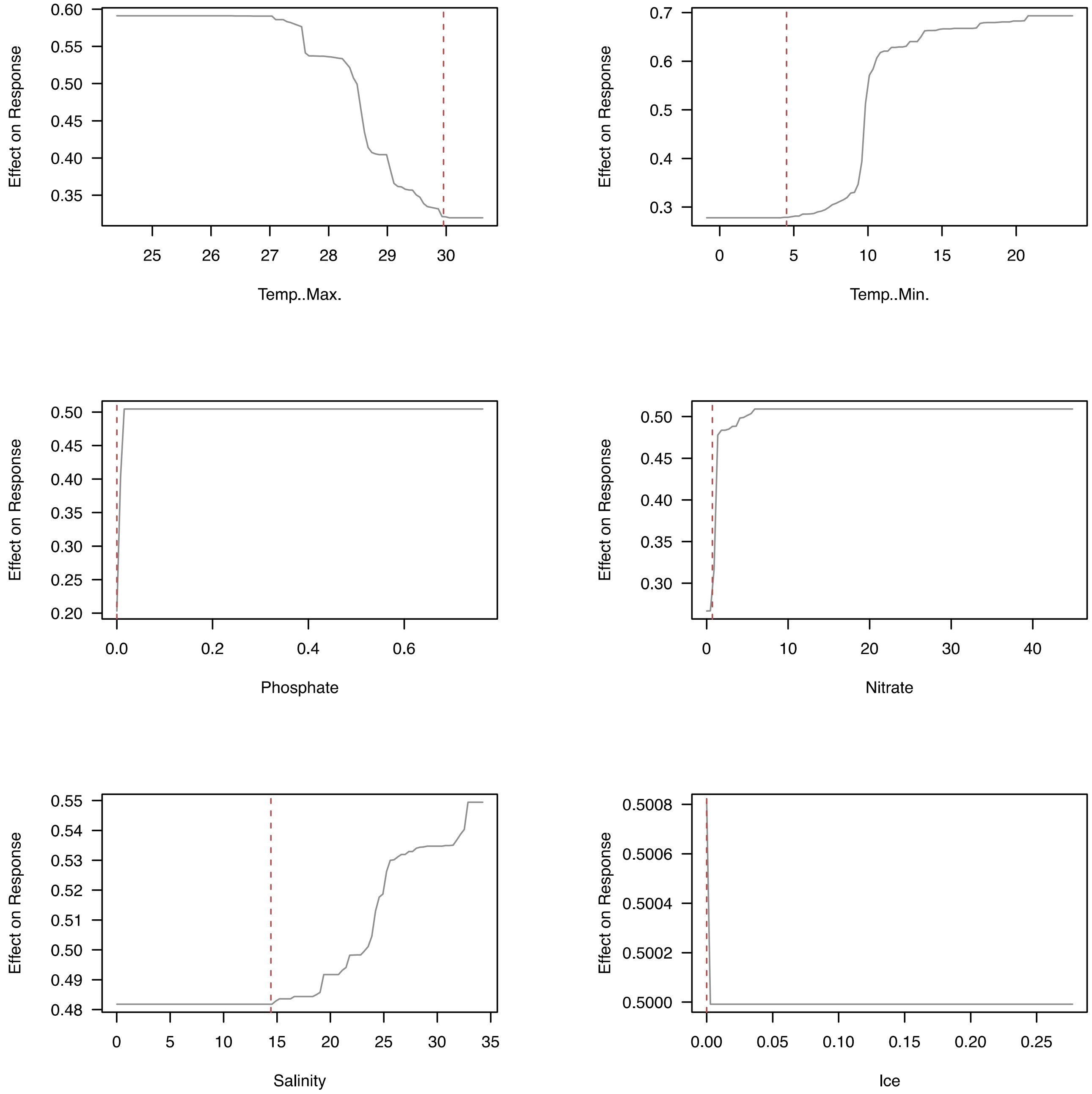

Figure S8. Partial dependency plots depicting the effect of each single predictor to the models developed for the southern genetic group.

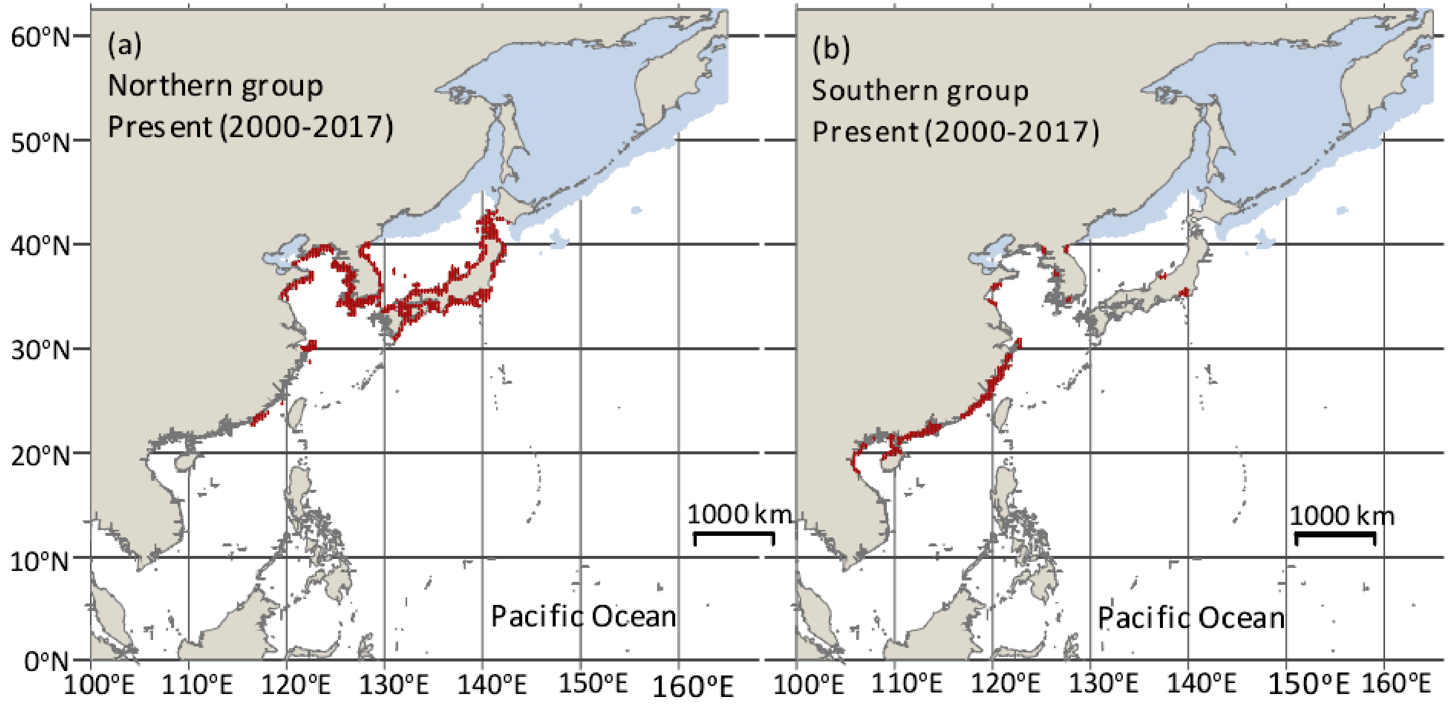

Figure S9. Potential distribution of the northern (a) and southern (b) genetic groups of *Sargassum thunbergii* predicted for the present. Light blue polygons depict seasonal sea ice and gray polygons depict land area.

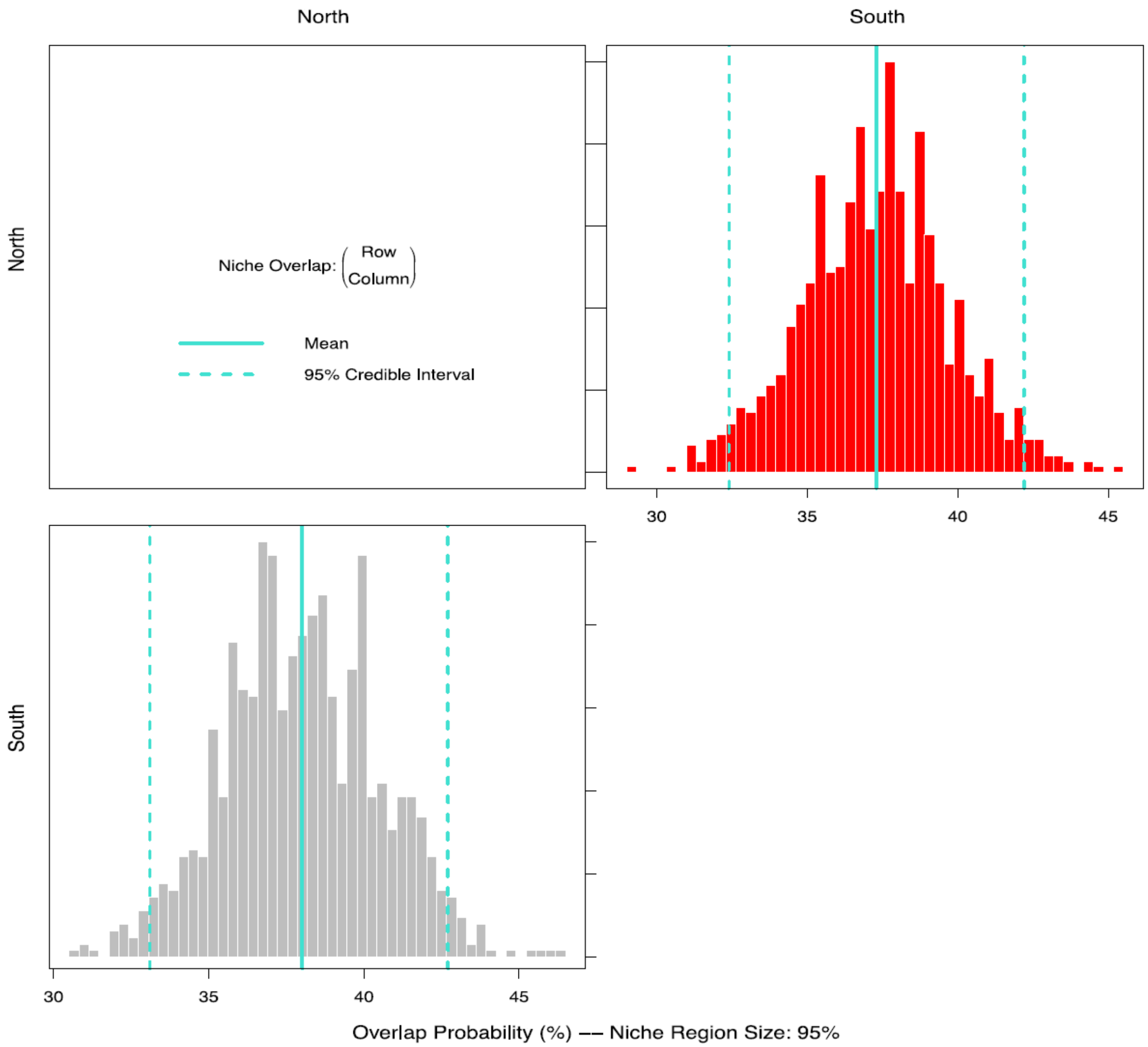

Figure S10. Probability (37.3%) of niche overlap between the northern and southern genetic groups of *S. thunbergii* and probability (38.1%) between the southern and the northern.

Supplemental Tables

Table S1. Historical occurrence records of *Sargassum thunbergii* in China.

Latitude	Longitude	Year of record	Full citation
39.54°N	122.97°E	2009	Yi et al. (2010) AFLP analysis of genetic diversity in six wild populations of *Sargassum thunbergii*. Journal of Fishery Sciences of China, 17:922-929 (In Chinese with English Abstract)
39.94°N	121.10°E	2008	Yi et al. (2010) AFLP analysis of genetic diversity in six wild populations of *Sargassum thunbergii*. Journal of Fishery Sciences of China, 17:922-929 (In Chinese with English Abstract)
39.26°N	122.63°E	2008	Yi et al. (2010) AFLP analysis of genetic diversity in six wild populations of *Sargassum thunbergii*. Journal of Fishery Sciences of China, 17:922-929 (In Chinese with English Abstract)
38.82°N	121.33°E	2008	Yi et al. (2010) AFLP analysis of genetic diversity in six wild populations of *Sargassum thunbergii*. Journal of Fishery Sciences of China, 17:922-929 (In Chinese with English Abstract)
39.92°N	119.72°E	2014	Li et al. (2014) Preliminary studies on the nitrogen and phosphorus absorption capability of seaweeds. Hebei Fisheries, 241:1-11 (In Chinese with English Abstract)
36.78°N	121.15°E	2008	Wang et al. (2009) AFLP analysis in populations of *Sargassum thunbergii* along China coast. Biotechnology Bulletin, Suppl. 277-279 (In Chinese with English Abstract)
35.35°N	119.42°E	1990	Liu et al. (1995) Preliminary study on the benthic algae in the intertidal region of muguan island. Transactions of Oceanology and Limnology, 1:80-86 (In Chinese with English Abstract)
28.68°N	121.43°E	2011	Quan et al. (2014) Growth and carbon sequestration rate of wild *Sargassum thunbergii* from different sources. Chinese Agricultural Science Bulletin, 30:79-82 (In Chinese with English Abstract)
27.51°N	121.08°E	2011	Quan et al. (2014) Growth and carbon sequestration rate of wild *Sargassum thunbergii* from different sources. Chinese Agricultural Science Bulletin, 30:79-82 (In Chinese with English Abstract)

24.40°N	118.35°E	1992	Chen et al. (1994) Antibacterial and antifungal activity of ten red and brown algae from Fujian coast. Journal of Fujian Normal University (Natural Science), 10:75-79 (In Chinese with English Abstract)
25.30°N	119.46°E	1989	Zheng Y and Chen ZH (1993) The seasonal growth and reproduction of *Sargassum thunbergii* (Phaeophyta) in Pingtan Island, Fujian province. Journal of Fujian Normal University (Natural Science), 9:81-85 (In Chinese with English Abstract)
23.42°N	117.25°E	1986	Yu et al. (1990) Studies on polysaccharides from algae. Journal of Shantou University (Natural Science). 5:55-60 (In Chinese with English Abstract)
23.35°N	117.13°E	1984	Zhang et al. (1990) A preliminary study on the ecology of the intertidal zone along the coast of southern Fujian province, China. Journal of Xiamen Fisheries College, 12:27-34 (In Chinese with English Abstract)

Table S2. Prior distributions of parameters for each scenario and posterior parameter values for scenario 3. The unit of time is generation, and the generation time of *S. thunbergii* is about 1-2 years.

Parameter	Prior distribution	Posterior parameter estimation		
		Median	Mode	95% credible interval
N1: population size in northern group	U(10, 10^5)	12600	5210	2640-59300
N2: population size in southern group	U(10, 10^4)	3700	4060	1330-6210
NA: population size in most recent ancestor	U(10, 10^6)	--	--	--
t1: Time of divergence (post-LGM)	U(10, 18000)	4790	3200	1400-11200
t2: Time of divergence (pre-LGM)	U(21000, 10^5)	--	--	--
μ: mean mutation rate per generation for SSR	U(10^{-4}, 10^{-3})	1.55×10^{-4}	1.00×10^{-4}	1.02×10^{-4}-5.24×10^{-4}

U, uniform distribution

Table S3. Pairwise F_{ST} differentiation between the 35 *Sargassum thunbergii* populations estimated using 11 microsatellite loci. Light blue and yellow colours represent $F_{ST} \geq 0.5$ and $0.25 \leq F_{ST} < 0.5$, respectively.

	1	2	3	4	5	6	7	8	9	10	11	12	13	14	15	16	17	18
1. Muroran, Hokkaido, Japan	0.000																	
2. Onagawa Bay, Miyagi, Japan	0.352	0.000																
3. Tateyama Bay, Chiba, Japan	0.609	0.641	0.000															
4. Chita, Aichi, Japan	0.534	0.511	0.618	0.000														
5. Shakotan, Hokkaido, Japan	0.300	0.379	0.547	0.458	0.000													
6. Esashi, Hokkaido, Japan	0.243	0.316	0.486	0.415	0.093	0.000												
7. Ama, Shimane, Japan	0.267	0.250	0.428	0.366	0.185	0.123	0.000											
8. Sokcho, Gangwondo, Korea	0.273	0.381	0.651	0.537	0.333	0.289	0.253	0.000										
9. Gauido, Chungcheongnamdo, Korea	0.152	0.372	0.644	0.573	0.371	0.288	0.284	0.346	0.000									
10. Taean, Chungcheongnamdo,Korea	0.128	0.323	0.591	0.504	0.282	0.225	0.237	0.298	0.159	0.000								
11. Yeongsan, Jeollanamdo, Korea	0.233	0.496	0.759	0.704	0.460	0.372	0.378	0.485	0.131	0.246	0.000							
12. Jodo, Jeollanamdo, Korea	0.193	0.431	0.667	0.606	0.335	0.292	0.315	0.359	0.220	0.180	0.253	0.000						
13. Jeju Island, Jejudo, Korea	0.316	0.268	0.534	0.428	0.200	0.158	0.180	0.324	0.372	0.285	0.462	0.371	0.000					
14. Dongbang, Liaoning, China	0.273	0.351	0.590	0.547	0.256	0.223	0.229	0.399	0.291	0.251	0.334	0.278	0.300	0.000				
15. Yingzuishi, Liaoning, China	0.234	0.310	0.517	0.461	0.196	0.172	0.188	0.355	0.252	0.196	0.324	0.271	0.235	0.058	0.000			
16. Yazishi, Liaoning, China	0.291	0.325	0.542	0.499	0.233	0.218	0.195	0.355	0.310	0.250	0.363	0.299	0.278	0.063	0.068	0.000		
17. Lvshun, Liaoning, China	0.213	0.287	0.563	0.521	0.292	0.236	0.226	0.346	0.231	0.191	0.283	0.250	0.298	0.113	0.119	0.135	0.000	
18. Beihuangcheng Isl., Yantai,China	0.246	0.353	0.559	0.510	0.255	0.210	0.234	0.387	0.292	0.232	0.308	0.257	0.286	0.085	0.064	0.108	0.128	0.000
19. Daqin Island, Yantai, China	0.259	0.306	0.537	0.499	0.274	0.224	0.221	0.347	0.283	0.253	0.308	0.266	0.286	0.093	0.087	0.111	0.106	0.041
20. Changdao Island, Yantai, China	0.303	0.372	0.595	0.553	0.298	0.266	0.269	0.384	0.345	0.298	0.379	0.318	0.312	0.113	0.097	0.113	0.166	0.096
21. Yantai Univeristy, Yantai, China	0.361	0.397	0.705	0.615	0.354	0.300	0.282	0.477	0.391	0.354	0.488	0.383	0.348	0.150	0.154	0.153	0.247	0.182
22. Jiming Island, Weihai, China	0.198	0.299	0.503	0.445	0.165	0.143	0.182	0.288	0.224	0.202	0.293	0.266	0.193	0.149	0.107	0.145	0.138	0.138
23. Xiaoshi Island, Weihai, China	0.287	0.376	0.651	0.582	0.353	0.274	0.307	0.428	0.322	0.272	0.391	0.358	0.303	0.195	0.164	0.250	0.200	0.160
24. Yueliang Bay, Weihai, China	0.293	0.356	0.624	0.536	0.281	0.233	0.240	0.414	0.329	0.286	0.413	0.319	0.290	0.123	0.104	0.130	0.194	0.113
25. Ailian Bay, Weihai, China	0.245	0.310	0.535	0.476	0.272	0.207	0.222	0.322	0.272	0.234	0.319	0.337	0.260	0.160	0.110	0.131	0.166	0.098
26. Chengshantou, Weihai, China	0.215	0.338	0.528	0.487	0.260	0.219	0.244	0.372	0.247	0.211	0.296	0.263	0.269	0.188	0.119	0.181	0.125	0.124
27. Badaguan, Qingdao, China	0.306	0.426	0.592	0.550	0.262	0.230	0.282	0.437	0.341	0.274	0.396	0.269	0.304	0.192	0.137	0.212	0.198	0.145
28. Shengsi, Zhoushan, China	0.470	0.522	0.686	0.631	0.382	0.369	0.346	0.466	0.491	0.483	0.587	0.521	0.390	0.393	0.330	0.348	0.400	0.374
29. Dongtou, Wenzhou, China	0.329	0.365	0.520	0.487	0.235	0.229	0.204	0.334	0.330	0.326	0.405	0.370	0.278	0.200	0.172	0.174	0.233	0.230
30. Longchuanjiao, Wenzhou, China	0.393	0.447	0.584	0.529	0.307	0.300	0.268	0.433	0.395	0.388	0.486	0.421	0.355	0.292	0.231	0.253	0.306	0.271
31. Sanpanwei, Wenzhou, China	0.430	0.472	0.630	0.581	0.339	0.326	0.293	0.451	0.440	0.430	0.529	0.463	0.362	0.323	0.267	0.283	0.344	0.316
32. Zhuyu Island, Wenzhou, China	0.440	0.463	0.619	0.572	0.350	0.331	0.293	0.446	0.445	0.427	0.535	0.467	0.365	0.338	0.280	0.290	0.357	0.336
33. Huangqi, Liangjiang, Fuzhou, China	0.448	0.495	0.656	0.597	0.343	0.343	0.308	0.457	0.473	0.444	0.560	0.474	0.351	0.362	0.302	0.313	0.389	0.341
34. Nanri Island, Putian, China	0.398	0.495	0.651	0.603	0.302	0.290	0.289	0.445	0.431	0.411	0.506	0.422	0.330	0.307	0.265	0.280	0.355	0.295
35. Meizhou Island, Putian, China	0.468	0.499	0.707	0.651	0.423	0.393	0.339	0.466	0.494	0.475	0.579	0.517	0.411	0.364	0.327	0.308	0.378	0.355

	19	20	21	22	23	24	25	26	27	28	29	30	31	32	33	34	35
1. Muroran, Hokkaido, Japan																	
2. Onagawa Bay, Miyagi, Japan																	
3. Tateyama Bay, Chiba, Japan																	
4. Chita, Aichi, Japan																	
5. Shakotan, Hokkaido, Japan																	
6. Esashi, Hokkaido, Japan																	
7. Ama, Shimane, Japan																	
8. Sokcho, Gangwondo, Korea																	
9. Gauido, Chungcheongnamdo, Korea																	
10. Taean, Chungcheongnamdo, Korea																	
11. Yeongsan, Jeollanamdo, Korea																	
12. Jodo, Jeollanamdo, Korea																	
13. Jeju Island, Jejudo, Korea																	
14. Dongbang, Liaoning, China																	
15. Yingzuishi, Liaoning, China																	
16. Yazishi, Liaoning, China																	
17. Lvshun, Liaoning, China																	
18. Beihuangcheng Island, Yantai, China																	
19. Daqin Island, Yantai, China	0.000																
20. Changdao Island, Yantai, China	0.082	0.000															
21. Yantai Univeristy, Yantai, China	0.220	0.226	0.000														
22. Jiming Island, Weihai, China	0.153	0.204	0.232	0.000													
23. Xiaoshi Island, Weihai, China	0.158	0.229	0.318	0.157	0.000												
24. Yueliang Bay, Weihai, China	0.162	0.178	0.025	0.169	0.237	0.000											
25. Ailian Bay, Weihai, China	0.104	0.139	0.247	0.104	0.128	0.176	0.000										
26. Chengshantou, Weihai, China	0.138	0.172	0.273	0.097	0.205	0.201	0.148	0.000									
27. Badaguan, Qingdao, China	0.151	0.200	0.349	0.176	0.240	0.262	0.227	0.138	0.000								
28. Shengsi, Zhoushan, China	0.342	0.350	0.508	0.279	0.452	0.440	0.322	0.355	0.428	0.000							
29. Dongtou, Wenzhou, China	0.199	0.216	0.317	0.155	0.310	0.274	0.193	0.235	0.291	0.128	0.000						
30. Longchuanjiao, Wenzhou, China	0.252	0.291	0.387	0.239	0.395	0.326	0.268	0.271	0.334	0.130	0.086	0.000					
31. Sanpanwei, Wenzhou, China	0.287	0.304	0.432	0.262	0.425	0.376	0.298	0.314	0.381	0.074	0.070	0.042	0.000				
32. Zhuyu Island, Wenzhou, China	0.301	0.309	0.449	0.286	0.436	0.394	0.302	0.329	0.391	0.124	0.068	0.063	0.034	0.000			
33. Huangqi, Liangjiang, Fuzhou, China	0.329	0.348	0.449	0.277	0.448	0.388	0.329	0.353	0.411	0.148	0.149	0.158	0.115	0.164	0.000		
34. Nanri Island, Putian, China	0.301	0.304	0.410	0.239	0.405	0.347	0.298	0.315	0.363	0.196	0.177	0.195	0.162	0.223	0.106	0.000	
35. Meizhou Island, Putian, China	0.313	0.315	0.477	0.319	0.464	0.419	0.311	0.373	0.449	0.161	0.163	0.186	0.139	0.181	0.117	0.169	0.000

Table S4. Pairwise Jost' *D* differentiation between the 35 *Sargassum thunbergii* populations estimated using 11 microsatellite loci. Light blue and yellow colours represent F_{ST} ≥0.75 and 0.5≤F_{ST}<0.75, respectively.

	1	2	3	4	5	6	7	8	9	10	11	12	13	14	15	16	17	18
1. Muroran, Hokkaido, Japan	0.000																	
2. Onagawa Bay, Miyagi, Japan	0.573	0.000																
3. Tateyama Bay, Chiba, Japan	0.914	0.781	0.000															
4. Chita, Aichi, Japan	0.839	0.590	0.355	0.000														
5. Shakotan, Hokkaido, Japan	0.545	0.656	0.722	0.625	0.000													
6. Esashi, Hokkaido, Japan	0.531	0.642	0.774	0.702	0.134	0.000												
7. Ama, Shimane, Japan	0.623	0.470	0.632	0.587	0.358	0.288	0.000											
8. Sokcho, Gangwondo, Korea	0.378	0.515	0.792	0.638	0.515	0.552	0.464	0.000										
9. Gauido, Chungcheongnamdo, Korea	0.175	0.536	0.868	0.824	0.670	0.589	0.592	0.467	0.000									
10. Taean, Chungcheongnamdo,Korea	0.160	0.518	0.885	0.773	0.512	0.492	0.537	0.445	0.189	0.000								
11. Yeongsan, Jeollanamdo, Korea	0.224	0.608	0.820	0.887	0.681	0.625	0.663	0.565	0.088	0.250	0.000							
12. Jodo, Jeollanamdo, Korea	0.230	0.639	0.857	0.856	0.522	0.561	0.647	0.455	0.240	0.215	0.195	0.000						
13. Jeju Island, Jejudo, Korea	0.689	0.446	0.809	0.647	0.357	0.351	0.420	0.577	0.776	0.611	0.801	0.716	0.000					
14. Dongbang, Liaoning, China	0.462	0.554	0.804	0.850	0.419	0.463	0.490	0.667	0.437	0.421	0.373	0.380	0.618	0.000				
15. Yingzuishi, Liaoning, China	0.455	0.565	0.776	0.760	0.350	0.403	0.454	0.678	0.436	0.367	0.450	0.453	0.533	0.076	0.000			
16. Yazishi, Liaoning, China	0.545	0.530	0.726	0.761	0.396	0.482	0.422	0.595	0.518	0.452	0.464	0.458	0.594	0.075	0.100	0.000		
17. Lvshun, Liaoning, China	0.339	0.427	0.761	0.801	0.535	0.519	0.501	0.552	0.327	0.301	0.305	0.341	0.638	0.149	0.195	0.201	0.000	
18. Beihuangcheng Isl., Yantai,China	0.408	0.576	0.734	0.757	0.430	0.438	0.519	0.654	0.451	0.387	0.341	0.348	0.594	0.103	0.088	0.151	0.180	0.000
19. Daqin Island, Yantai, China	0.448	0.469	0.688	0.738	0.487	0.487	0.486	0.556	0.440	0.448	0.347	0.374	0.604	0.118	0.134	0.158	0.145	0.040
20. Changdao Island, Yantai, China	0.506	0.568	0.752	0.806	0.496	0.554	0.579	0.584	0.532	0.510	0.425	0.434	0.617	0.135	0.137	0.145	0.228	0.112
21. Yantai Univeristy, Yantai, China	0.521	0.489	0.846	0.756	0.510	0.525	0.493	0.664	0.506	0.523	0.496	0.450	0.579	0.150	0.194	0.168	0.302	0.199
22. Jiming Island, Weihai, China	0.378	0.562	0.776	0.750	0.287	0.332	0.457	0.518	0.386	0.401	0.407	0.463	0.429	0.261	0.211	0.269	0.246	0.242
23. Xiaoshi Island, Weihai, China	0.439	0.543	0.876	0.839	0.611	0.546	0.663	0.662	0.449	0.420	0.416	0.490	0.559	0.253	0.249	0.380	0.274	0.202
24. Yueliang Bay, Weihai, China	0.470	0.517	0.821	0.730	0.445	0.453	0.483	0.649	0.484	0.470	0.479	0.426	0.542	0.147	0.148	0.170	0.272	0.136
25. Ailian Bay, Weihai, China	0.446	0.520	0.755	0.736	0.522	0.473	0.527	0.538	0.449	0.432	0.401	0.579	0.569	0.251	0.192	0.209	0.275	0.140
26. Chengshantou, Weihai, China	0.353	0.559	0.683	0.721	0.461	0.482	0.572	0.636	0.369	0.354	0.337	0.377	0.564	0.293	0.200	0.300	0.182	0.178
27. Badaguan, Qingdao, China	0.511	0.712	0.735	0.787	0.406	0.454	0.615	0.726	0.520	0.449	0.455	0.337	0.590	0.262	0.209	0.322	0.286	0.188
28. Shengsi, Zhoushan, China	0.770	0.755	0.680	0.733	0.539	0.678	0.631	0.585	0.711	0.844	0.675	0.734	0.651	0.539	0.510	0.481	0.582	0.514
29. Dongtou, Wenzhou, China	0.666	0.644	0.676	0.736	0.405	0.520	0.454	0.547	0.578	0.677	0.566	0.645	0.603	0.323	0.323	0.289	0.412	0.398
30. Longchuanjiao, Wenzhou, China	0.699	0.710	0.629	0.650	0.473	0.607	0.527	0.653	0.605	0.707	0.596	0.624	0.690	0.427	0.378	0.376	0.476	0.394
31. Sanpanwei, Wenzhou, China	0.744	0.713	0.663	0.713	0.506	0.631	0.551	0.636	0.664	0.775	0.634	0.671	0.654	0.451	0.423	0.403	0.520	0.450
32. Zhuyu Island, Wenzhou, China	0.798	0.708	0.662	0.712	0.548	0.662	0.568	0.645	0.698	0.787	0.672	0.705	0.678	0.499	0.466	0.427	0.568	0.508
33. Huangqi, Liangjiang, Fuzhou, China	0.776	0.751	0.701	0.726	0.496	0.659	0.577	0.629	0.734	0.791	0.686	0.678	0.601	0.522	0.489	0.449	0.612	0.490
34. Nanri Island, Putian, China	0.662	0.795	0.747	0.801	0.427	0.537	0.548	0.633	0.651	0.726	0.589	0.577	0.575	0.426	0.426	0.403	0.556	0.415
35. Meizhou Island, Putian, China	0.741	0.662	0.708	0.763	0.625	0.729	0.594	0.564	0.697	0.793	0.625	0.699	0.691	0.461	0.489	0.387	0.513	0.458

	19	20	21	22	23	24	25	26	27	28	29	30	31	32	33	34	35
1. Muroran, Hokkaido, Japan																	
2. Onagawa Bay, Miyagi, Japan																	
3. Tateyama Bay, Chiba, Japan																	
4. Chita, Aichi, Japan																	
5. Shakotan, Hokkaido, Japan																	
6. Esashi, Hokkaido, Japan																	
7. Ama, Shimane, Japan																	
8. Sokcho, Gangwondo, Korea																	
9. Gauido, Chungcheongnamdo, Korea																	
10. Taean, Chungcheongnamdo, Korea																	
11. Yeongsan, Jeollanamdo, Korea																	
12. Jodo, Jeollanamdo, Korea																	
13. Jeju Island, Jejudo, Korea																	
14. Dongbang, Liaoning, China																	
15. Yingzuishi, Liaoning, China																	
16. Yazishi, Liaoning, China																	
17. Lvshun, Liaoning, China																	
18. Beihuangcheng Island, Yantai, China																	
19. Daqin Island, Yantai, China	0.000																
20. Changdao Island, Yantai, China	0.094	0.000															
21. Yantai Univeristy, Yantai, China	0.260	0.239	0.000														
22. Jiming Island, Weihai, China	0.281	0.366	0.346	0.000													
23. Xiaoshi Island, Weihai, China	0.203	0.293	0.363	0.247	0.000												
24. Yueliang Bay, Weihai, China	0.218	0.219	0.013	0.283	0.301	0.000											
25. Ailian Bay, Weihai, China	0.154	0.198	0.330	0.187	0.169	0.261	0.000										
26. Chengshantou, Weihai, China	0.206	0.244	0.359	0.163	0.290	0.294	0.243	0.000									
27. Badaguan, Qingdao, China	0.203	0.259	0.444	0.302	0.311	0.366	0.371	0.185	0.000								
28. Shengsi, Zhoushan, China	0.453	0.417	0.605	0.419	0.599	0.596	0.449	0.493	0.578	0.000							
29. Dongtou, Wenzhou, China	0.335	0.336	0.454	0.298	0.524	0.455	0.348	0.428	0.505	0.127	0.000						
30. Longchuanjiao, Wenzhou, China	0.364	0.397	0.476	0.416	0.598	0.459	0.429	0.411	0.486	0.098	0.097	0.000					
31. Sanpanwei, Wenzhou, China	0.399	0.385	0.520	0.433	0.617	0.523	0.457	0.466	0.545	0.044	0.069	0.027	0.000				
32. Zhuyu Island, Wenzhou, China	0.439	0.407	0.574	0.504	0.667	0.583	0.478	0.514	0.584	0.086	0.068	0.048	0.019	0.000			
33. Huangqi, Liangjiang, Fuzhou, China	0.471	0.456	0.534	0.454	0.654	0.532	0.512	0.538	0.597	0.099	0.168	0.140	0.085	0.137	0.000		
34. Nanri Island, Putian, China	0.435	0.392	0.482	0.387	0.579	0.468	0.464	0.475	0.511	0.151	0.220	0.194	0.138	0.215	0.078	0.000	
35. Meizhou Island, Putian, China	0.385	0.343	0.515	0.494	0.608	0.529	0.414	0.517	0.609	0.094	0.167	0.150	0.093	0.134	0.072	0.121	0.000

Table S5. Variable contribution (%) and physiological tolerance limits inferred with Boosted Regression Trees considering within-taxon niche structure into two main genetic groups of *S. thunbergii* in the Northwest Pacific.

	**North**		**South**	
	**Contribution**	**Tolerance limit**	**Contribution**	**Tolerance limit**
Min. ocean temp.	0	-	**32.30**	4.39 ^{0}C
Max. ocean temp.	**26.17**	28.75 ^{0}C	**31.88**	29.93 ^{0}C
Ice thickness	**72.53**	0.02 m	0	-
Salinity	0.87	22.01	4.174	14.45
Nitrates	0.08	0.320 mmol . m^{-3}	**14.48**	0.553 mmol . m^{-3}
Phosphates	0.35	0.017 mmol . m^{-3}	**17.14**	0.015 mmol . m^{-3}
